# Regulation of metastatic potential by drug repurposing and mitochondrial targeting in colorectal cancer cells

**DOI:** 10.1186/s12885-024-12064-5

**Published:** 2024-03-08

**Authors:** Shashank Mathur, Pransu Srivastava, Anubhav Srivastava, Neeraj Kumar Rai, Sabiya Abbas, Ashok kumar, Meenakshi Tiwari, Lokendra Kumar Sharma

**Affiliations:** 1 Department of Molecular Medicine and Biotechnology, Sanjay Gandhi Post Graduate Institute of Medical Sciences, Rae Bareli Road, (U.P.) Lucknow, 226014 India; 2 Department of Surgical Gastroenterology, Sanjay Gandhi Post Graduate Institute of Medical Sciences, Rae Bareli Road, (U.P.) Lucknow, 226014 India; 3 Department of Biochemistry, All India Institute of Medical Sciences, Patna Bihar, 801507 India

**Keywords:** Mitochondria, Mitochondrial complex I, Oxidative stress, Metastasis, Mitochondrial antibiotics

## Abstract

**Background:**

Increased mitochondrial activities contributing to cancer cell proliferation, invasion, and metastasis have been reported in different cancers; however, studies on the therapeutic targeting of mitochondria in regulating cell proliferation and invasiveness are limited. Because mitochondria are believed to have evolved through bacterial invasion in mammalian cells, antibiotics could provide an alternative approach to target mitochondria, especially in cancers with increased mitochondrial activities. In this study, we investigated the therapeutic potential of bacteriostatic antibiotics in regulating the growth potential of colorectal cancer (CRC) cells, which differ in their metastatic potential and mitochondrial functions.

**Methods:**

A combination of viability, cell migration, and spheroid formation assays was used to measure the effect on metastatic potential. The effect on mitochondrial mechanisms was investigated by measuring mitochondrial DNA copy number by qPCR, biogenesis (by qPCR and immunoblotting), and functions by measuring reactive oxygen species, membrane potential, and ATP using standard methods. In addition, the effect on assembly and activities of respiratory chain (RC) complexes was determined using blue native gel electrophoresis and in-gel assays, respectively). Changes in metastatic and cell death signaling were measured by immunoblotting with specific marker proteins and compared between CRC cells.

**Results:**

Both tigecycline and tetracycline effectively reduced the viability, migration, and spheroid-forming capacity of highly metastatic CRC cells. This increased sensitivity was attributed to reduced mtDNA content, mitochondrial biogenesis, ATP content, membrane potential, and increased oxidative stress. Specifically, complex I assembly and activity were significantly inhibited by these antibiotics in high-metastatic cells. Significant down-regulation in the expression of mitochondrial-mediated survival pathways, such as phospho-AKT, cMYC, phospho-SRC, and phospho-FAK, and upregulation in cell death (apoptosis and autophagy) were observed, which contributed to the enhanced sensitivity of highly metastatic CRC cells toward these antibiotics. In addition, the combined treatment of the CRC chemotherapeutic agent oxaliplatin with tigecycline/tetracycline at physiological concentrations effectively sensitized these cells at early time points.

**Conclusion:**

Altogether, our study reports that bacterial antibiotics, such as tigecycline and tetracycline, target mitochondrial functions specifically mitochondrial complex I architecture and activity and would be useful in combination with cancer chemotherapeutics for high metastatic conditions.

**Supplementary Information:**

The online version contains supplementary material available at 10.1186/s12885-024-12064-5.

## Introduction

Colorectal cancer (CRC) is the third most commonly diagnosed cancer and second in cancer-related death worldwide as per GLOBOCAN [1], with an increasing trend in developing nations, including India [2]. Although treatment mainly relies on surgical removal and/or chemotherapy, recent developments in targeted therapies, have also improved patient survival [3]. However, tumor metastasis, recurrence, and chemo-resistance limit the effectiveness of such therapies. Therefore, alternate approaches are required to regulate cancer cell growth and identify suitable therapeutic combinations for the treatment of metastatic cancers.

Warburg proposed that cancer cells preferentially use glycolysis for their bioenergetic requirements because of impairments in mitochondrial respiration [4]. However, later it was redefined that mitochondrial metabolism and reactive oxygen species (ROS) generation are essential for cancer cell proliferation and play an important role in different stages of cancer progression, including invasiveness and metastatic transition [5, 6]. Therefore, targeting mitochondria and their associated mechanisms is taking center stage in cancer research to investigate alternative therapeutic approaches [7].

Because mitochondria are thought to evolve through bacterial invasion in human cells, antibacterial agents such as antibiotics could be exploited to inhibit mitochondrial machinery in cancer cells. Anticancer antibiotics such as doxorubicin, bleomycin, mitomycin, dactinomycin, and macrolides, which exert their effects by nonspecifically targeting cancer cell DNA, are being used to treat various malignant tumors [8]; however they may also cause toxicity to normal tissues. Bacterial antibiotics that inhibit protein synthesis, such as tetracycline and their chemical derivatives, are well tolerated in normal human cells and have been reported to have antiproliferative effects in certain cancer types [9, 10]. Some of these types of antibiotics can also selectively bind to mitochondrial ribosomal components, inhibit protein translation through t-RNA binding, transpeptidation, or initiation steps, and inhibit cellular growth [11]. For example, FDA-approved bacteriostatic agents such as erythromycin and chloramphenicol; bind to the large ribosomal subunit (50s), and the tetracycline and tigecycline class of antibiotics bind to the small ribosomal subunit (30s) of bacteria. Their corresponding binding to mitochondrial large ribosomal (39s) and small (28s) subunits could be of great importance in investigating their effect on mitochondrial activities [12]. Although, the antiproliferative effects of these antibiotics have been investigated in different cancer cells, their effects on mitochondrial biogenesis, respiratory complex architecture, and functions remain unclear. The inhibitory effect of antibiotics in human CRC, especially in metastatic conditions, which are linked to higher mitochondrial activities, is largely unknown [13, 14]. Previously, we reported that increased mitochondrial biogenesis along with upregulated mitochondrial complex I (C-I) activity confers survival, invasiveness, and proliferation in highly metastatic CRC cells and that pharmacological inhibition of C-I could regulate the proliferation of these cells [13]. In the present study, we aimed to investigate the therapeutic potential of different antibiotics on the sensitivity of low- and high-metastatic CRC cells and to understand the regulation of mitochondria after antibiotic treatment in these cells.

## Material and methods

### Cell lines

Established human colorectal cancer cell lines with different metastatic potentials -HT29 and -HCT15 (low); and -HCT116 and Colo205 (high) were procured from the national repository at the National Centre for Cell Sciences (NCCS) Pune, India. NCCS Pune provides authentic cell lines verified by the short tandem repeat (STR) profiling method, and early passage cells were used for performing experiments in this study. Cell lines were grown in RPMI 1640 medium supplemented with 10% fetal bovine serum and antibiotic–antimycotic solution (1%), all purchased from Gibco^TM^ (Thermo Scientific Inc.), and maintained at 37°C with 95% humidity and 5% CO2 in an incubator.

### Cell viability assay

Cells were seeded in a 48-well cell culture plate at 10,000 cells/well in triplicate, incubated at 37°C for 24h, and treated with different concentrations of antibiotics (Chloramphenicol/ Erythromycin/Linezolid/Tigecycline/Tetracycline: all from Sigma) for the next 24/48 h. For therapeutic agent/combination treatment, cells were treated with oxaliplatin (Sigma) with or without antibiotics for given time intervals. After treatment, cells were collected by trypsinization and stained with trypan blue (0.4% in PBS), followed by live/dead cell counting and viability in Countess^TM^ 3 cell counter (Thermo Scientific Inc.). In parallel, bright-field images were captured at total 100x magnification using an inverted microscope (Olympus IX73). The IC_50_ values were calculated for the concentration that inhibited cell growth by 50 percent using the standard liner regression curve in excel sheet (Microsoft Inc).

### Transwell assay

The metastatic potentials of cells before and after treatment were measured on the basis of their chemoattractant movement through extracellular matrix invasion following a previous protocol [13]. In brief, an equal number of cells (5 x10^5^) were seeded in triplicate in 8-µm pore-sized cell culture inserts ((BD Labware; BD Biosciences) with serum-free media +/- Tig/Tet antibiotics. Cell inserts were positioned in 24-well plates containing media containing 10% FBS, followed by incubation for 12 h at 37°C in a 5% CO_2_ incubator. The insert was removed, and cells that migrated toward the opposite side of the insert were fixed in methanol and stained with crystal violet blue in the methanol– water system. The stained cells were visualized and images were captured using a bright-field inverted microscope at total 100x magnification. The total number of migrated stained cells was counted and calculated as the relative migration units between untreated and treated cells.

### Spheroid formation assay

In vitro 3D spheroid generation from CRC cells was performed by modifying a previously described protocol [15]. Briefly, CRC cells were seeded in non-adherent conditions (0.9% Agarose overlay) in 96-well plates at a density of 5000 cells/well, and a total of 100µl of medium supplemented with or without Tg/Tet. After 24 h of incubation, fresh media supplemented with 0.2% collagen was added to facilitate spheroid formation. Spheroid growth and morphological changes were regularly monitored and imaged for the next 3 days using a bright-field inverted microscope. These spheroids were carefully lifted and collected for trypsinization to generate a single-cell suspension and for viability by trypan blue staining, as mentioned earlier.

### Mitochondrial DNA copy number measurement

The relative mt-DNA copy number was measured by quantitative PCR (qPCR) using mitochondrial-tRNA leucine 1 and nuclear 18-s rRNA gene- specific primers, following a previously published protocol [13]. Nuclear 18-s RNA gene was used as an internal reference gene for normalizing mtND1 gene amplification in each case. TB green-based relative amplification was performed following the manufacturer’s instructions (Takara Bio Inc.) and run on a 7900HT Fast Real-Time PCR System (Applied Biosystem). The qPCR conditions were as follows: initial denaturation for 3 min at 95 °C, followed by 40 cycles at 95 °C for 15 sec; and 60 °C for 60 sec. The relative expression of mtDNA versus nDNA was calculated by 2^−ΔCt^ (ΔCt = Ct mtDNA - Ct nDNA) following a previously published method [16], and fold changes were calculated compared with untreated cells as the respective control.

### Reverse transcription– quantitative PCR (RT-qPCR)

For gene expression studies, total RNA was isolated from untreated/treated cells using the RNeasy kit (Qiagen) and quantitated using NanoDrop™ spectrophotometers (Thermo Scientific Inc). One microgram of total RNA was reverse transcribed into cDNA using the Revert Aid^TM^ Reverse Transcription kit following the manufacturer’s instructions (Invitrogen). Subsequently, qPCR of target genes (PGC1-α/NRF1/NRF2/TFAM) and normalization control (β-actin) was performed using fast SYBR Green Master mix following similar PCR conditions as reported previously [13]. The relative gene expression of target genes was normalized to β-actin expression (reference gene) using $2^{-\triangle \text{C}_\text{T}}$ method [16].

### Oxidative stress measurement

Total cellular ROS and mitochondrial superoxide levels were measured using fluorescent indicator probes such as 2′,7′-dichlorodihydrofluorescein diacetate (H_2_-DCFDA) and MitoSOX™ Red respectively as per the manufacturer’s instructions (Thermo Fisher Scientific, Inc.). Briefly, after 24/48 h of antibiotic treatment, cells were collected and equal numbers of cells (1×10^4^), were incubated separately with H_2_DCFDA (10 µM)/MitoSOX™ Red (5 µM) at 37°C for the next 15 min. The cells were then washed, centrifuged briefly at room temperature, and suspended in PBS. The fluorescence intensity was measured using an HT-BioTek fluorescence plate reader at corresponding Ex/Em values of 495/529 nm for H_2_DCFDA or 510/580 nm for MitoSOX™ Red at 37°C. Fluorescence intensity was normalized to the total cellular count and presented as a relative fold change compared with the non-treated control as 1.

### Mitochondrial membrane potential measurements

Mitochondrial membrane potential was measured by staining non-treated/treated cells with JC1 dye following the manufacturer’s instructions (Invitrogen) and a previously published protocol [17]. An equal number of cells was incubated with JC1 (2 μM) for 15 min followed by quantification of J aggregates with red fluorescence at Ex/Em: 550/600nm and monomer form with green fluorescence at Ex/Em: 485/535 nm in a BioTek Synergy HT Multi-detection Microplate reader. All experiments were performed in triplicate at least 2-3 times, and the ratio of red to green fluorescence was calculated and presented as the relative fold change to non-treated cells.

### ATP measurement

Total ATP content was measured using the ATPlite Luminescence Assay System according to the manufacturer’s instructions (Perkin –Elmer Inc.). Briefly, total 1×10^4^ cells were incubated with antibiotics for 24/48 h in a 96-well white bottom plate (in triplicate). After treatment, ATPlite buffer was added to the cells for further 10 min incubation in the dark, and luminescence was measured immediately using a BioTek Synergy HT Multi-detection Microplate reader. The luminescence was adjusted for the total cell count and calculated as the relative fold change compared with that of the untreated cells.

### Blue native polyacrylamide gel electrophoresis (BN-PAGE)

Mitochondrial ETC complex assembly was assessed using BN-PAGE according to a previously published protocol [13]. After treatment with antibiotics for 24 h, cells were harvested by trypsinization and collected by centrifugation at room temperature. The pellet was suspended in 0.7 ml of ice-cold IB buffer (Tris Cl-pH: 7.4; 1 mM EDTA, 250 mM sucrose) containing protease inhibitor cocktail (Sigma-Aldrich). Cells were lysed using a Dounce homogenizer by applying approximately 50-60 strokes at 4°C and enriched for mitochondria via differential centrifugation at 4°C (initial spin at 500 × g for 5 min, followed by re-spun of supernatant to pellet the mitochondria at 10,000 × g for 5 min). The obtained mitochondrial pellet was washed and re-suspended in a final protein concentration of 2–5 mg/ml in IB buffer. Mitochondrial pellets were solubilized using digitonin solution (8 mg digitonin/mg of protein in HB buffer without EDTA) to achieve optimal solubility of mitochondrial RC complexes. The samples were incubated on ice for 20 min in 5% Coomassie blue solution at a v/v ratio of 1:30. A total of 80 µg protein/sample was then subjected to 3%–12% Native PAGE (Novex Bis-Tris gel) for 4 h at 80 V and 4°C in the NativePAGE™ Running Buffer (Invitrogen; Thermo Fisher Scientific, Inc.) to resolve the mitochondrial complexes. The resulting gels were stained with Bio-safe Coomassie R-250 for 30 min, and densitometry analysis of band intensities was performed using ImageJ software. In parallel, a similar aliquot of the corresponding sample was run on a denaturing gel for western blotting of the loading control VDAC as a normalizing control for individual complex intensities.

### Measurements of respiratory complex activities

The activities of different respiratory complexes (C-I, V and IV) were measured following previously published protocols [18, 19]. In brief, equal concentrations of mitochondrial preparation (40µg each) were loaded in sets on BN-PAGE, and different complexes were resolved as mentioned earlier. The resulting gels were used for in-gel activity assays of individual complexes. For RC-I activity, BN-PAGE gel strips were incubated in freshly prepared complex I activity buffer (2.5 mg/ml of NTB and NADH (0.1mg/ml) in 2 mM Tris/HCl, pH 7.4) for 15-30 min. Subsequently, the gel was transferred to a fixing solution (Water: Methanol: Acetic Acid, 50:40:10) to stop the NADH-NTB reduction reaction (until dark blue/purple color develops). For RC-V activity measurement, the gel strip was pre-incubated in 20 mM Tris-Cl buffer (pH-8.2) for 1 h and subsequently replaced with complex V assay buffer (34 mM Tris,200mM Glycine,14 mM MgSO4, and 0.2% Pb(NO3)2 (pH 7.8). Freshly prepared ATP (8mM) was added to initiate the reaction and kept overnight until a white precipitate of lead nitrate accumulated over the complex V band. The gel was transferred to milliQ water to stop the reaction. For RC-IV activity, the gel strip was incubated with freshly prepared complex IV assay buffer [cytochrome c (10mg), Catalase (20µg/ml), sucrose (750mg), and diaminobenzidine (5 mg) in 10 ml of 50 mM phosphate buffer (pH=7.4)] for 30 min until a dark brown residue formed, and the reaction was stopped by transferring the gel to fixing solution. All resulting images after in-gel assays were scanned immediately after the assays in the gel documentation system (BioRad). For equal loading of mitochondrial preparation, the same amount of protein was used in parallel for SDS-PAGE and immunoblotting with VDAC protein. The corresponding band intensities of specific complexes were analyzed by densitometry using ImageJ software, normalized with VDAC, and compared with the non-treated control.

### Western blotting

Protein expression was assessed by western blotting following a previously published protocol [13]. In brief, protein was extracted from the cell pellet using RIPA buffer (Hi Media Inc.), and the concentration was determined using a BCA Protein assay kit (Pierce; Thermo Fisher Scientific, Inc.). A total of 30 µg protein for each sample was electrophoresed on 12% SDS-PAGE at 100 V for 2 h, and the resolved proteins were transferred onto a PVDF membrane. After blocking in 5% bovine serum albumin (BSA) in TBS-0.5% Tween-20 (TBS-T) for 1 h at room temperature, the membrane was incubated with primary antibodies at a dilution of 1:1,000 overnight at 4°C following the manufacturer’s instructions. To detect more than one target protein on a single blot, the same membrane was cut into one or more pieces before antibody hybridization based on the molecular weight of the target protein. All antibodies (except PGC-1α, TFAM and β-actin from Santa Cruz Inc.) were procured from Cell Signaling Technology Inc. The membrane was washed three times with TBS-T and then incubated with HRP-labeled secondary antibody at a 1:5,000 dilution. Protein bands were developed using the clarity MaxTM Western ECL substrate (Bio-Rad Laboratories, Inc.) and imaged using a ChemiDoc Imaging system (Bio-Rad Laboratories, Inc.). Densitometry analysis was performed on each western blot using NIH-Image J software to quantify the bands. The individual values were normalized to their corresponding actin levels and presented as fold changes relative to the untreated control. Each experiment was repeated at least 2-3 times. The figures show representative images with almost similar results for the same experiments and the corresponding relative fold changes Images of the un-cropped original western blots are included in the supplementary file.

### Statistical analysis

GraphPad Prism Software (version 9, San Diego, CA, USA) was used to prepare comparative graphs between the control (untreated) and treated groups. Graphical data are represented as mean ± SEM of experiments that were repeated at least 2-3 times with ≥3 replicates for each group. Morphological images were representative of three or more separate experiments with similar results. Significant statistical differences were calculated using unpaired Student’s t-test or one-way analysis of variance (ANOVA) followed by Dunnett's post hoc test for comparing treatment and control groups or Tukey’s test for multiple group comparisons. A p-value below 0.05 was deemed to represent a statistically significant difference.

## Results

### Differential sensitivity of antibiotics to CRC cell survival

**Fig. 1:**
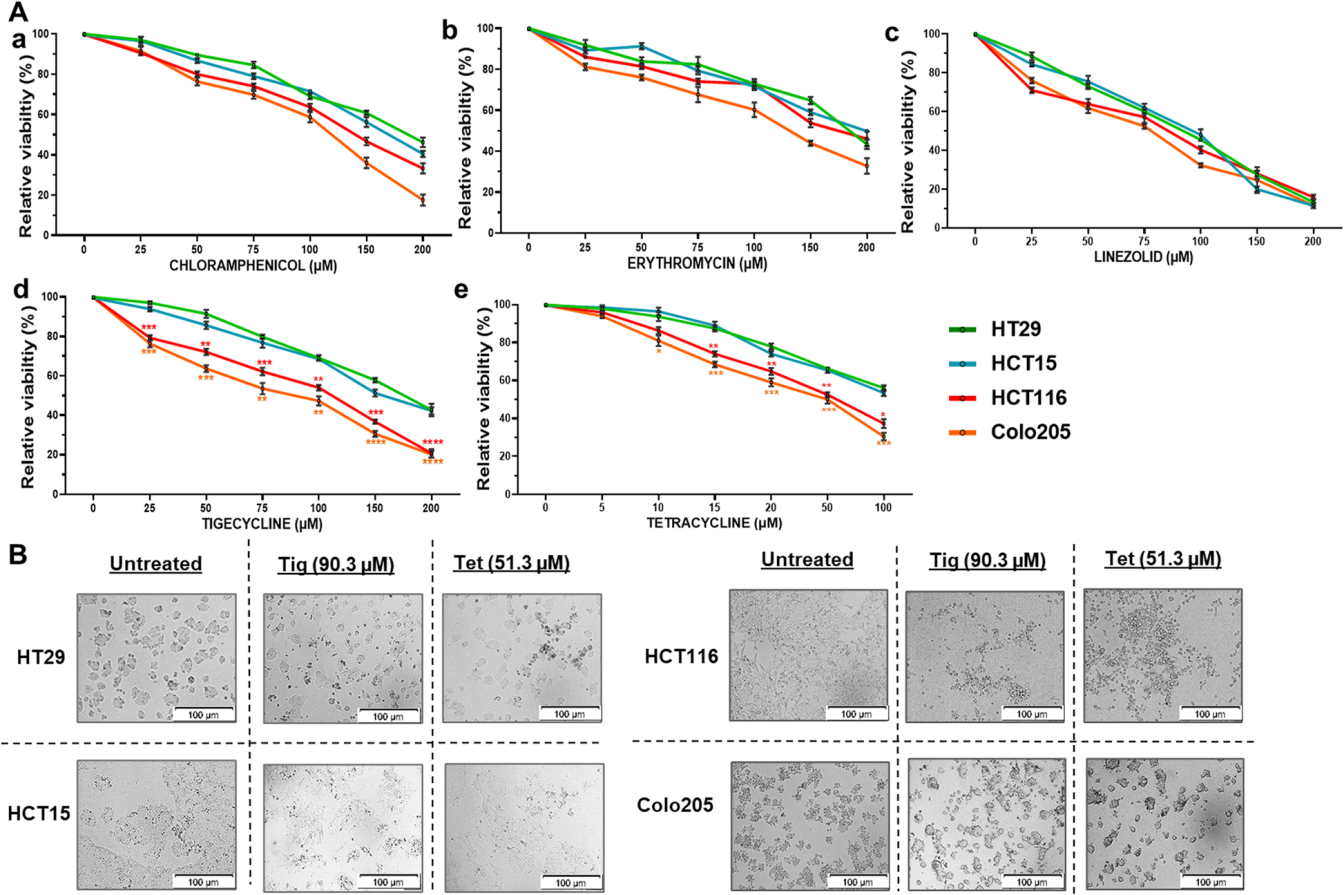
Differential sensitivity of CRC cells to various antibiotics. A A pair of low (HT29 and HCT15) and high (HCT116 and Colo205) metastatic cells were treated with different classes of antibiotics at varying concentrations (0-200 µM) for 48h and changes in cell viability were measured by trypan blue staining. Graphs represent the relative percentage viability of CRC cells (low and high metastatic cells) after treatment with a chloramphenicol, b erythromycin, c linezolid, d tigecycline, and e tetracycline. Data represent the mean ± standard deviation (SD) of viability relative to their respective non-treated controls. Student’s t-test was used to calculate the significant difference in the viability of CRC cells, compared to the percentage viability of HT29 cells (one of the low metastatic cells) at the indicated concentrations. *P<0.05, **P<0.01 and ***P<0.001, ****P<0.0001.B After treatment with IC_50_ concentrations of tigecycline and tetracycline for 48 h, bright field images of CRC cells were captured using an Olympus IX73 microscope. Representative images at total 100x magnification (100µm scale bar) are shown to indicate morphological differences at IC_50_ concentrations, especially the dead rounded cells in high metastatic cells (right panel) compared with low metastatic CRC cells (left panel)

To investigate the effect of different classes of antibiotics on the survival and proliferation of CRC cells, a pair of low (HT29 and HCT15) and high metastatic (HCT116 and Colo205) CRC cells, known for differences in metastatic potential and mitochondrial functions, was used [13]. Cells were treated with varying concentrations (ranging from 0 to 200 µM) of antibiotics for 48 h, and changes in viability were measured using the trypan blue exclusion assay. As shown in Figure 1A, a general trend of reduced viability was observed among all cell lines after antibiotic treatment. No significant difference in the viability of low and high metastatic cells was observed upon chloramphenicol/erythromycin/linezolid treatment (Fig. 1Aa-c). However, upon tigecycline/tetracycline treatment, high metastatic cells showed a significant dose-dependent reduction in viability from 25µM of tigecycline (Tig) and 15µM of tetracycline (Tet) up to higher concentration of 200µM and 100µM respectively at 48h in comparison with low metastatic CRC cells (Fig. 1A:d-e). The analysis of half-maximal inhibitory concentration (IC_50_) resulted in significantly lower IC_50_ values (Mean ± SD: Tig IC_50_ = 90.3 ± 23.06 µM, Tet IC_50_= 51.5±19 µM) for high metastatic (HCT116/Colo205) cells than low metastatic (HT29/HCT15) cells (Tig IC_50_=196.35 ±17.4 µM, Tet IC_50_= 102.5 ± 15.8 µM) (Supplementary Table 1). Furthermore, typical morphological features of cell death, such as rounding of cells and detachment from the surface, were more prominent in high metastatic cells at their respective IC_50_ concentrations than in non-treated controls (Fig. 1B). However, at these concentrations, low metastatic cells were relatively healthy with better viability. These results indicated that Tet/Tig treatment significantly inhibited the viability of high metastatic cells at much lower concentrations than low metastatic cells.

### Tig/Tet treatment reduces metastatic and spheroid formation in highly metastatic cells

**Fig. 2:**
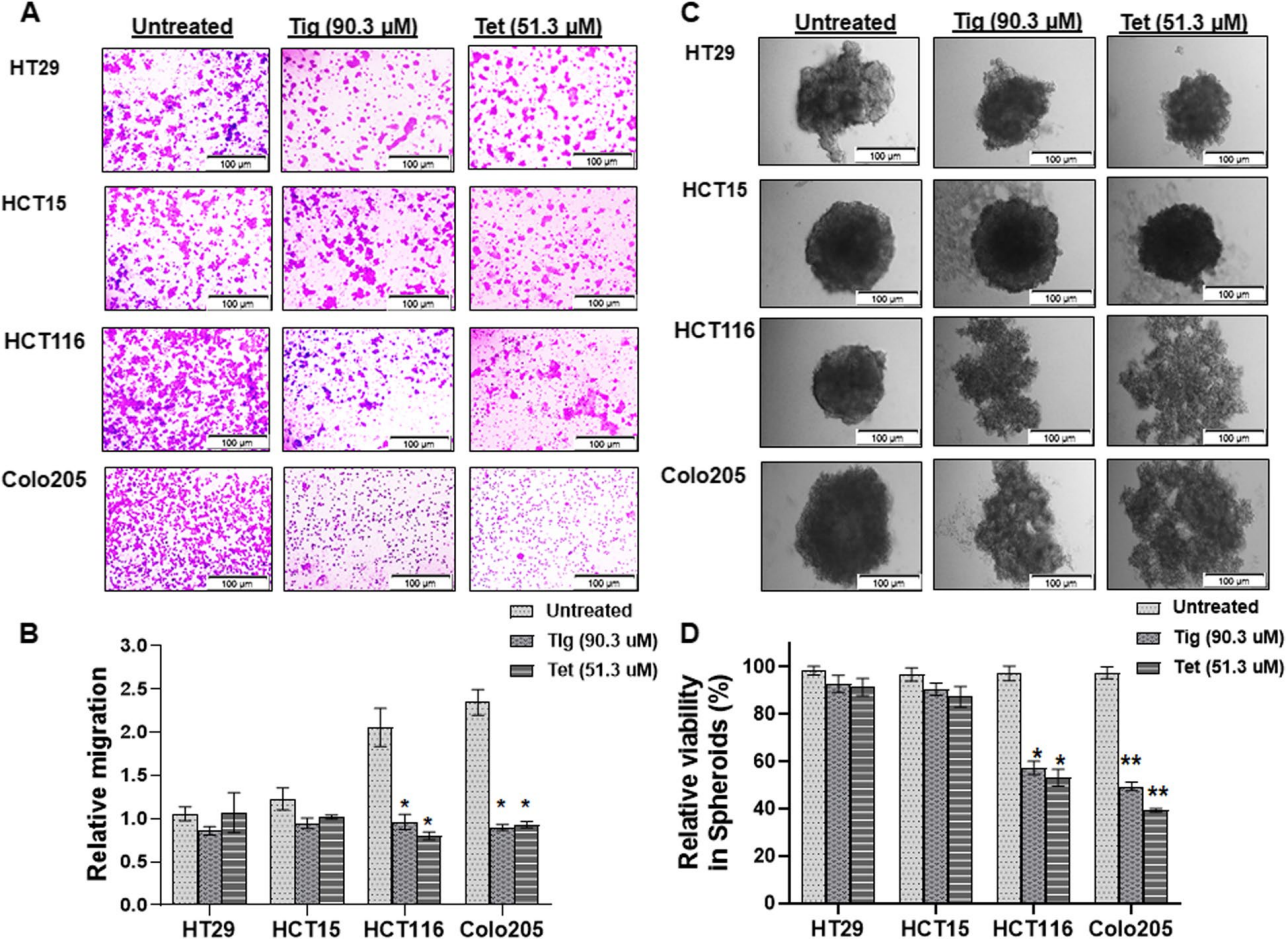
Effect of tigecycline/tetracycline on in vitro metastatic potential and spheroid formation. **A-B** In vitro metastatic potential was measured using transwell migration of CRC cells following treatment with Tig/Tet for 12 h. Cells migrated through trans-well membranes were stained with crystal violet and visualized under a microscope at total 100x magnification (100µm scale bar). **A** Representative images of migrated cells are shown. **B** Stained cells were counted and represented as relative migration units. **C-D** Cells were treated with Tig/Tet under low attachment conditions for 24h and allowed to form spheroids subsequently for the next 4–5 days. **C** Representative images of spheroids showing morphological changes on the 5^th^ day were acquired under a bright-field microscope at total 100x magnification (100µm scale bar). **D** Viability of single cell suspension derived from spheroids after trypsnization and counting by trypan blue staining. Results are representative of three experimental replicates for each cell line, repeated at least 2–3 times, and values represent mean± SD. *P<0.05, **P<0.01

The differential sensitivity of Tig/Tet in CRC cells was investigated for their effect on in vitro metastatic potential and proliferation using transwell and three-dimensional (3D) spheroid formation assays, respectively. The trans-well assay is a well-established in vitro method for testing the metastatic potential of cancer cells by measuring the chemotactic movement of cells through the trans-well membrane. Cells were seeded with Tig/Tet in serum-free media in a cell culture insert, which was placed in a 24-well plate supplemented with complete media. As shown in Fig 2A, the number of cells that migrated from serum-free media toward complete media (to the lower side of the trans well membrane) after Tig/Tet treatment was significantly lower in HCT116/Colo205 cells than in non-treated control or similarly treated HT29/HCT15 cells. Quantitative measurement of migration resulted in an overall reduction of 0.46- to 0.40-fold in HCT116/Colo205 cells after Tig/Tet treatment relative to that in the untreated control (Fig 2B). No significant difference in migration was observed in HT29/HCT15 cells after Tig/Tet treatment.

Alternatively, to investigate the effect of Tig/Tet treatment on growth potential, the effect on 3D spheroid formation of CRC cells was measured. 3D spheroids are useful in-vitro tumor models that closely mimic tumor architecture and are considered to be a better pre-clinical drug screening platform than monolayer cells. Therefore, the ability of CRC cells to generate compact and round spheroids under non-adherent conditions and sustain cellular viability upon Tig/tet treatment was evaluated using the spheroid formation assay and trypan blue exclusion assay, respectively. As shown in Fig 2C, after Tig/Tet treatment for 24h, the 3D spheroids in HCT116/Colo205 cells appeared loose, less compact, and more fragmented compared with their non-treated cells and/or HT29/HCT15 cells. Subsequently, single-cell suspensions derived from these spheroids resulted in a significant reduction in viability upon Tig/Tet treatment from (~35-60%) in HCT116/Colo205 compared with their non-treated controls (Fig 2D). However, the viability of HT29/HCT15 cells did not change significantly after similar treatment.

### Tig/Tet treatment alters mitochondrial content and biogenesis in CRC cells

**Fig. 3:**
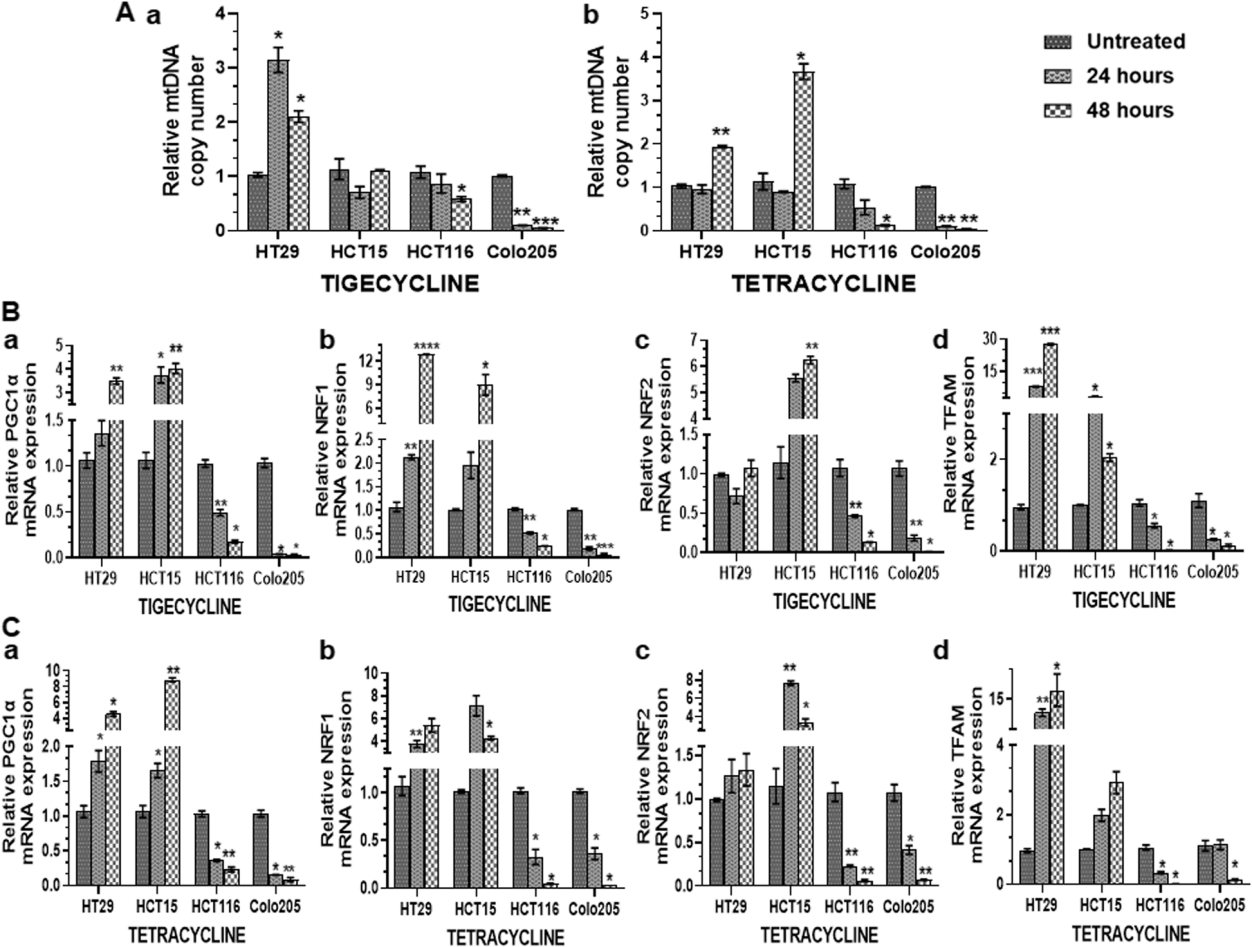
Differential effect of tigecycline/tetracycline on mtDNA levels and biogenesis. After treatment with Tig/Tet at respective IC_50_ concentrations for 24 and 48 h, total DNA and RNA were extracted for A measuring mtDNA copy number and B-C mRNA expression of mitochondrial biogenesis markers, respectively, by TB green-based qPCR. A Relative mtDNA copy number was determined by quantifying the mt-tRNA leucine 1 gene copy number and normalized with nuclear 18s rRNA copy number in different cells upon treatment with tigecycline Aa and tetracycline Ab. The cells were treated with B tigecycline and C tetracycline for 24 and 48h and the relative mRNA expression of mitochondrial biogenesis markers a PGC1-α, b NRF1, c NRF2, and d TFAM, normalized to β-actin expression, was evaluated by RT-qPCR. Data are the Mean ± SD of three replicates from a representative experiment normalized to the respective control. *P<0.05, **P<0.01 and ***P<0.001. Abbreviations: PGC1-α, peroxisome proliferator, activated receptor γ coactivator α; NRF, nuclear respiratory factor; TFAM, mitochondrial transcription factor A; mtDNA, mitochondrial DNA; qPCR, quantitative polymerase chain reaction; RT-reverse transcription

Given the evolutionary origin of mitochondria from bacteria, we further investigated the effect of Tig/Tet on mitochondrial content and biogenesis after 24/48 h treatment in different CRC cells. Changes in mitochondrial content were analyzed by measuring the copy number of the mtDNA-encoded tRNA leucine 1 gene and normalizing it with the nuclear 18s ribosomal RNA reference gene copy number. As shown in Fig 3A(a-b), both Tig and Tet treatment significantly inhibited mtDNA content in high-metastatic HCT116/Colo205 cells, especially at 48 h, compared with the non-treated controls. However, at 24 h, this inhibition was significant only in Colo205 cells. In contrast, low metastatic cells displayed an overall increasing trend in mtDNA content at 48h for both antibiotics (except for HCT15-Tig in Fig 3Aa). However, at 24h, the mtDNA content mostly remained unaltered in these cells, except for the increased mtDNA content in HT29-Tig (Fig 3Aa).

Because changes in mitochondrial content are driven by mitochondrial biogenesis, the effect of Tig/Tet treatment on the expression of mitochondrial biogenesis markers, such as PGC1-α, NRF1/2, and TFAM, was assessed. While PGC1-α is an upstream transcription factor, NRF1, NRF2, and TFAM are downstream factors in the mitochondrial biogenesis pathway. The gene expression of PGC1α, NRF1/2, and TFAM was quantitated by RT-qPCR in different cell lines after 24/48 h of Tig/Tet treatment. As shown in Fig. 3 (B and C), the expression of biogenesis markers was significantly downregulated in high-metastatic cells at both time points as well as for both Tig (Fig. 3B (a-d)) and Tet (Fig. 3C (a-d)) antibiotics (except no change in TFAM levels in Colo205-Tet at 24h, Fig 3Bd). Conversely, an increasing trend of upregulation of biogenesis markers was observed in low metastatic cells, which was significant mostly at 48h for both antibiotics (Fig 3B and C), with some exceptions of no change in NRF2 in HT29-Tig/Tet (Fig 3Bc and Cc) and TFAM in HCT15 Tet (Fig 3Cd) at 48 h.

The overall results clearly indicated that Tig/Tet treatment differentially altered mtDNA content and biogenesis in these cells. Specifically, antibiotic treatment reduced the mtDNA content and downregulated the expression of mitochondrial biogenesis markers in highly metastatic cells, which may contribute to their enhanced sensitivity to these mitochondrial antibiotics.

### Tig/Tet treatment inhibits mitochondrial function in CRC cells

The major function of mitochondria is to generate ATP by oxidative phosphorylation (OXPHOS), which is catalyzed by the transfer of electrons from NADH/FADH2 to oxygen via respiratory chain complexes (complex I to IV) and the movement of protons from the inter-membrane space to the matrix (via complex V) to generate ATP. In addition, electron leakage from RC complexes may generate mitochondrial superoxide radicals for the cytosolic reactive oxygen species (ROS) pool, which is regulated through the antioxidant defense system. However, under stress conditions, the enhanced level of ROS contributes to oxidative stress, which deregulates proliferative and survival pathways and may lead to disease progression. Because the investigation of these parameters could reflect mitochondrial functionality and indicate their contribution to cellular proliferation and death pathways, the effect of Tig/Tet on these different mitochondrial parameters was investigated.

**Fig. 4:**
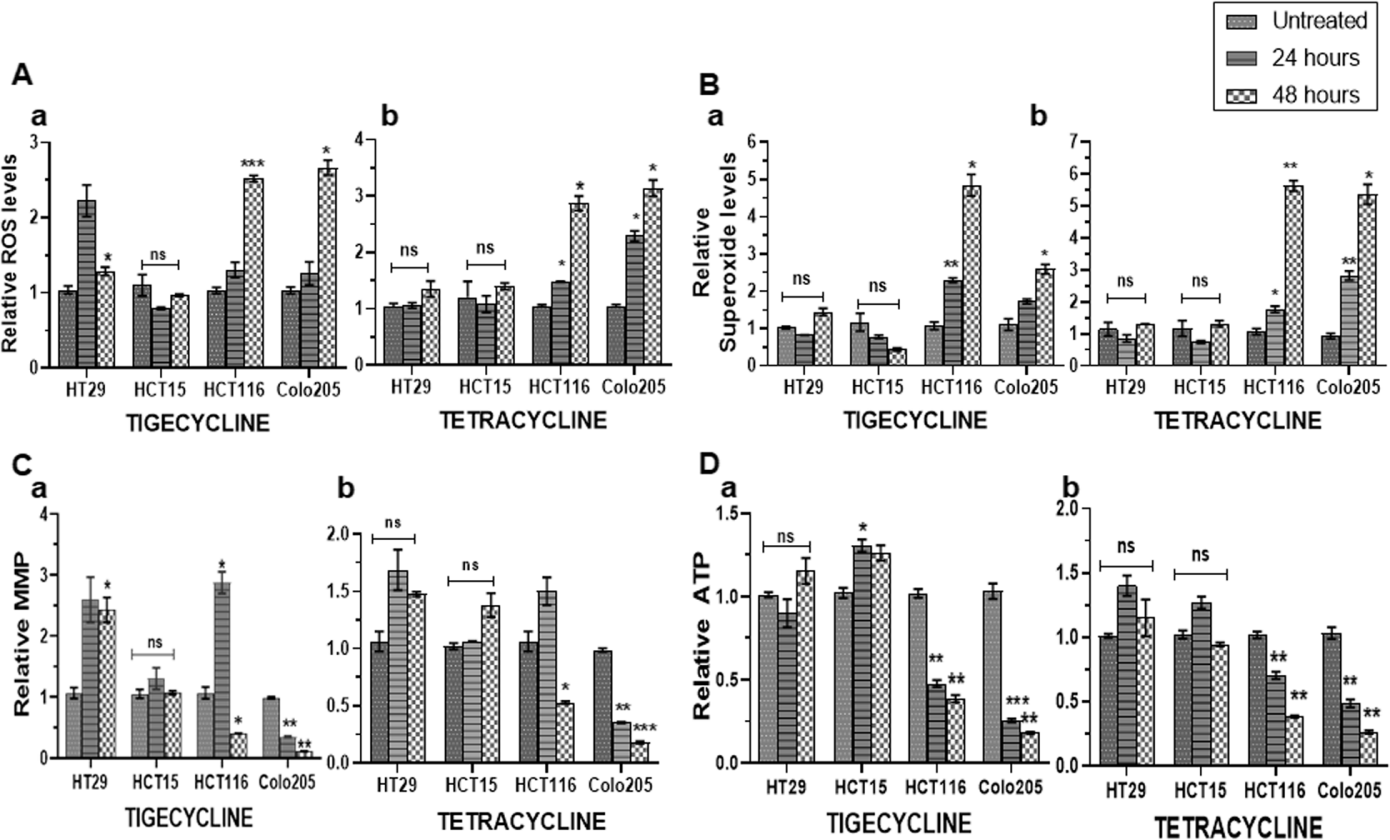
Effect of tigecycline/tetracycline treatment on mitochondrial function. Cells were treated with Tig/Tet for 24 and 48 h, and equal numbers of cells (10,000 each in triplicates) were used for analysis of mitochondrial parameters along with untreated controls. A Total ROS levels were quantified by staining of cells with 2′,7′-dichlorodihydrofluorescein diacetate (H_2_DCFDA), and B mitochondrial superoxide levels were measured by MitoSOX™ staining, followed by fluorescence plate reading. For both measurements, the cells were counterstain with nuclear stain Hoechst33342 for normalization. C Changes in mitochondrial membrane potential were measured by JC1 staining and quantifying the J aggregates red fluorescence at 590nm and normalized with nuclear stain Hoechst33342 readings in a plate reader. D Change in total cellular ATP content was measured in cells with a luciferase-based one-step ATP detection through luminescence and normalized with equal cell count. Relative levels of different parameters are shown individually in graphs for a Tig and b Tet. *P<0.05**P<0.01 and ***P<0.001 vs. Untreated. Abbreviations: ROS, reactive oxygen species; ATP, adenosine triphosphate; JC1, 5,5,6,6′-tetrachloro-1,1,3,3′-tetraethylbenzimi-dazoylcarbocyanine iodide

For oxidative stress measurement, total ROS and mitochondrial superoxide levels were measured using fluorescent indicator dyes such as H_2_DCFDA and MitoSox Red, respectively. The ROS indicator dye H_2_DCFDA undergoes oxidation to generate highly fluorescent 2′,7′-dichlorofluorescein (DCF) radicals, and MitoSox Red is selectively targeted to mitochondria and readily oxidized by mitochondrial superoxide. Both the final oxidized products can be quantitated at their respective fluorescence levels in a plate reader. As shown in Fig. 4A, a significantly higher level of ROS was observed in highly metastatic cells following treatment with Tig/Tet at 48 h. However, at 24 h, the response of Tig or Tet to ROS levels varied among different cell lines. For example, while Tig did not alter the ROS level in any of the cell lines at 24h, it increased ROS significantly in high metastatic cells at 48h (HCT116: 2.4 fold, Colo205: 2.5 fold), along with a small yet significant increase in HT-29 of low metastatic cells (Fig 4A-a). Compared with Tig, Tet treatment significantly increased ROS levels at both time points in high metastatic cells, including 2.78- and 3.04-fold in HCT116 and Colo205 cells, respectively, at 48 h (Fig 4A-b). Similarly, mitochondrial superoxide levels were also significantly increased in high-metastatic cells 48 h after Tig/Tet treatment (Fig 4B). Although superoxide levels did not change after Tig treatment at 24h in high metastatic cells (except HCT116), they were significantly increased at 48h in these cells (HCT116: 4.5 fold, Colo205: 2.3fold) (Fig 4B-a). Tet treatment significantly increased superoxide levels at both time points, including a > 5-fold increase in both high metastatic cells at 48h (Fig. 4B-b). Altogether, both antibiotics increased ROS and superoxide levels at 48 h in high metastatic cells; however, Tet triggered early oxidative stress at 24 h compared with Tig.

Because alterations in ROS production affect the mitochondrial membrane potential (MMP- ΔΨM) and vice versa, MMP levels were also determined using JC-1 staining. JC-1 dye is a lipophilic, cationic dye with green fluorescence that accumulates inside mitochondria in a concentration-dependent manner to form reversible complexes called J aggregates with red fluorescence. In healthy cells with a normal ΔΨM, the JC-1 accumulates in negatively charged mitochondria and fluorescent J-aggregates. Conversely, in unhealthy or apoptotic cells where mitochondria are less negative, JC-1 enters to a lesser degree, forms fewer J aggregates, and retains its monomeric form with original green fluorescence. As shown in Fig. 4C, JC1 aggregation was significantly decreased in highly metastatic cells 48 h after Tig/Tet treatment. However, individual cell lines of the low and high metastatic set differed in their response to Tig or Tet treatment. For example, Tig treatment significantly increased JC1 aggregation in one of the low-metastatic HT29 cells, but not in HCT15, at 48 h; however, no significant change was recorded in either of these cells at 24 h (Fig. 4C-a). Similarly, an increase in JC1 aggregation was also observed in high-metastatic HCT116 cells only after Tig treatment at 24 h, whereas it was significantly decreased in both cells at 48 h (0.554- and 0.125-fold in HCT116 and Colo205, respectively) (Fig. 4C-a). For Tet treatment, low metastatic cells did not show any significant change in JC1 aggregation at 24 or 48h; however, a significant decline was observed in high metastatic cells, especially at 48h (0.519- and 0.11-fold reduction in HCT116 and Colo205, respectively) (Fig. 4C-b). Overall, ΔΨM was significantly reduced upon Tig/Tet treatment in highly metastatic cells, especially at 48 h.

In addition, the total ATP content after Tig/Tet treatment was measured using ATPlite ™ One-Step assay, which was based on a firefly luciferase system. As shown in Fig. 4D, a significant depletion in ATP levels was observed after Tig (Fig. 4Da) or Tet (Fig 4Db) treatment at both 24 and 48 h in high metastatic cells, including an approximately 0.2-fold decrease at 48 h. There was no significant change in ATP levels after Tig/tet treatment in low metastatic cells, except for a small yet significant increase after Tig treatment in HCT15 cells at 24 h (Fig 4D-b).

Altogether, functional analysis of mitochondria upon Tig/Tet treatment in CRC cells suggested that the enhanced sensitivity of high metastatic cells could be due to enhanced oxidative stress, reduced membrane potential, and lower ATP levels.

### Tig/Tet treatment inhibits respiratory chain assembly and activity in high metastatic cells

**Fig. 5:**
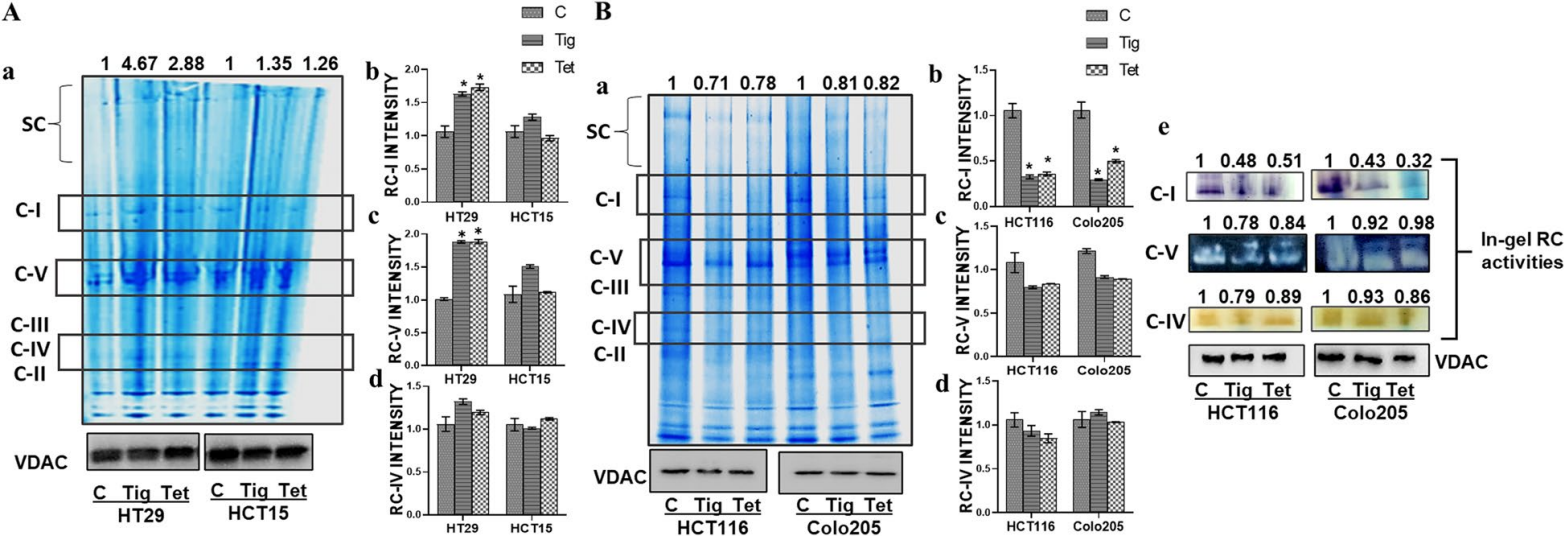
Analysis of RC assembly and activities after Tig/Tet treatment. Low and high metastatic CRC cells were treated with Tig/Tet for 24 h, and mitochondria were isolated for analyzing RC complex assembly by BN-PAGE and activities by in-gel assays as described in the Methods section. To ensure equal loading of samples, denaturing SDS-PAGE was performed in parallel, followed by immunoblotting with mitochondrial loading control VDAC. Results of Tig/Tet treatment in A low metastatic and B high metastatic cells are presented as a RC assembly showing Coomassie blue stained image and loading control VDAC blot; b-d relative fold change in band intensities of C-I b, C-V c and C-IV d as analyzed by densitometry. Band intensities of Coomassie-stained bands were normalized with the band intensity of corresponding VDAC values and are presented as fold change relative to non-treated control. (B-e) In addition, gel assay results of different RC complexes in high metastatic cells after treatment are shown. The values represent the fold change relative to the non-treated control. These experiments were performed 2–3 times, and the images are representative of one of the experiments with similar results. Abbreviations: RC, Respiratory complex; VDAC, Voltage-dependent anion channel; BN-PAGE, Blue native polyacrylamide gel electrophoresis

The functionality of mitochondria also depends on the respiratory chain (RC) architecture, which includes the assembly and activities of RC complexes. RC complexes, which are encoded by both mitochondrial and nuclear DNA, assemble and form supercomplexes required for the functional activity of RC. Thus, the effect of Tig/Tet treatment on RC assembly was investigated using BN-PAGE with mitochondrial preparations isolated from CRC cells after Tig/Tet treatment for 24 h. A standard assembled pattern of various RC complexes (ranging from 1MDa-100kDa) was visualized after Coomassie blue staining of the BN-PAGE gel followed by the quantitation of band intensities, which were normalized with VDAC loading control; run parallel in denaturing conditions for the same aliquot (Fig 5). An overall increase in RC complex proteins was observed in low metastatic HT29/HCT15 cells upon treatment (Fig 5Aa), and a decrease was observed in HCT116/Colo205 cells (Fig 5Ba). Upon measurement of the band intensity of individual complexes (mainly C-I/C-V/C-IV), in low metastatic cells, C-I and C-V intensities were higher in HT29 cells only (Fig 5A: b-c), whereas the intensity of C-IV did not change in any of the low metastatic cells after Tig/Tet treatment (Fig 5A: d). In contrast, in high metastatic cells, a significant decline in C– I intensity was observed (Fig 5B: b); however, the intensity of other complexes (C-V and IV) remained unchanged after Tig/Tet treatment (Fig 5B: c-d). Furthermore, this reduced intensity of C-I also resulted in a corresponding decrease in C-I activity in high metastatic cells after Tig/Tet treatment, as confirmed by the gel activity assay (Fig. 5B: e). The activities of the other complexes (C-V and C-IV) remained unchanged. Altogether, BN-PAGE analysis indicated that Tig/Tet treatment inhibited the assembly of the RC complex and decreased the assembly and activity of C-I in high metastatic cells.

### Tig/Tet treatment limits mitochondrial biogenesis and proliferative signaling and promotes apoptosis in high metastatic cells

Alteration in mitochondrial activities, including biogenesis, bioenergetics, and oxidative stress, could decide the fate of cells to proliferation or death. Therefore, to further understand the end effect of Tig/Tet treatment in CRC cells, changes in biogenesis, energy sensing, antioxidant defense, and proliferative and death signaling pathways were investigated. Cells were treated with Tg/Tet for 48h and the expression of different marker proteins, such as those for mitochondrial biogenesis (PGC1-α and TFAM), energy sensing (AMPK), proliferation (AKT and c-Myc), metastasis (p-FAK and p-SRC), and cell death (apoptosis: PARP and Caspase- 3 and autophagy: LC3B and p62) were investigated using immunoblotting methods.

**Fig. 6:**
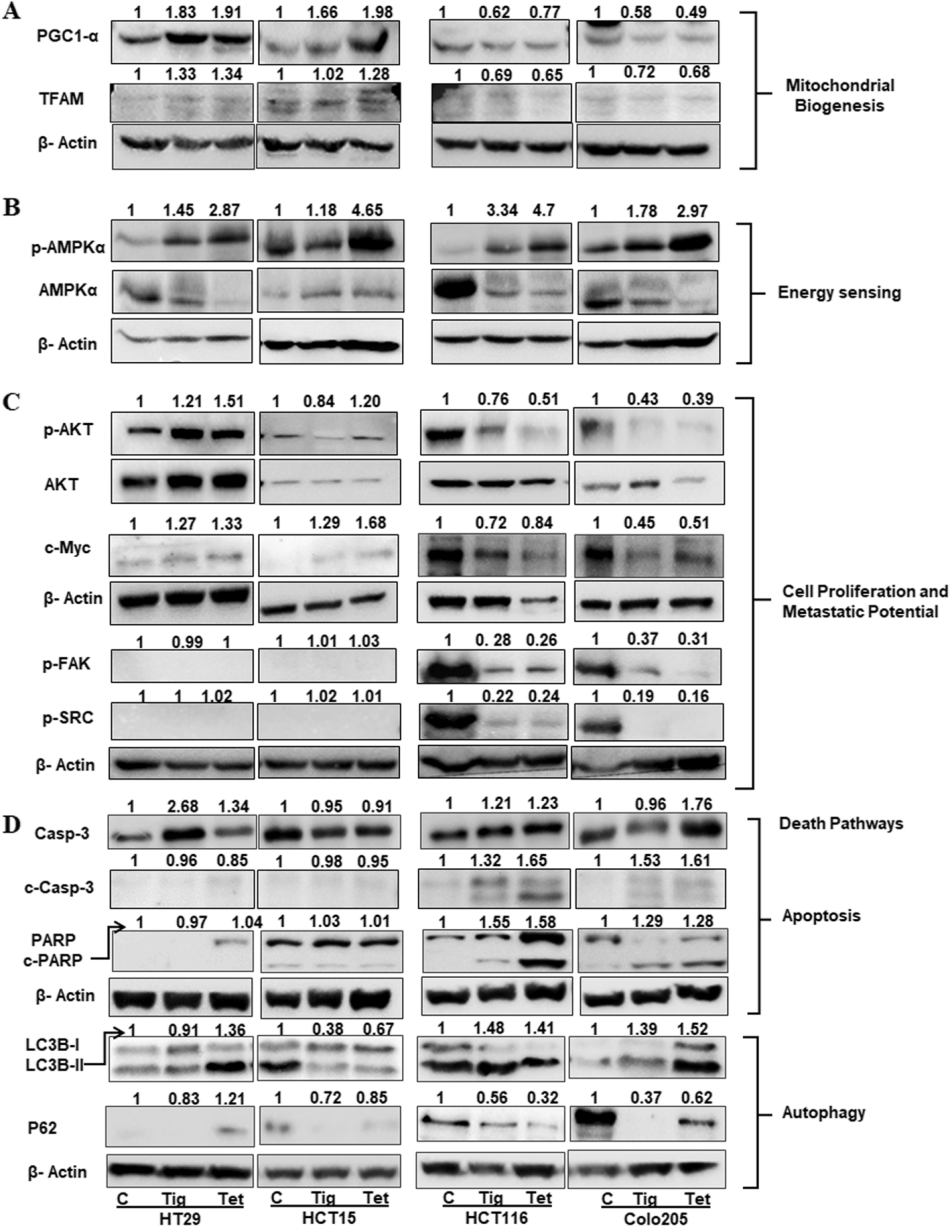
Analysis of cellular signaling pathways modulated by Tig/Tet treatment. Cells were treated with Tig/Tet for 48h and 30µg protein from cell lysate was used for immunoblotting with different marker proteins of cellular signaling pathways. Figure represents **A** PGC1-α and TFAM protein levels for mitochondrial biogenesis; **B** phospho-AMPK-α (Thr172) and AMPKα proteins for energy sensing; **C** phospho-AKT (Ser473), AKT, and cMyc expression for proliferative and phosphorylation of FAK and SRC protein levels for metastatic signaling; and **D** caspase-3 and PARP (c-Casp-3 and c-PARP), and LC3II and p62 marker protein expression for apoptosis and autophagy pathways, respectively. Band intensity was normalized with their corresponding β-actin band and presented as the relative fold change compared with non-treated controls. Each experiment was performed at least 2-3 times with similar results, and the images and corresponding values are representative of one of those experiments. Abbreviations: PGC1-α, peroxisome proliferator-activated receptor-gamma coactivator (PGC)-1alpha; TFAM, transcription factor A, mitochondrial; p-, phosphorylated; AMPK, adenosine monophosphate-activated protein kinase; AKT, protein kinase B; FAK, focal adhesion kinase, SRC, tyrosine-protein kinase Src; PARP, Poly (ADP-ribose) polymerases; LC3B, microtubule associated protein 1 light chain 3 beta

As shown in Fig 6A, a decrease in the protein expression of mitochondrial biogenesis markers such as PGC1α and TFAM was observed in high-metastatic cells upon treatment. Conversely, these factors were mostly upregulated in low metastatic cells after Tig/Tet treatment (except unchanged TFAM levels in HCT-15 Tig treatment condition). As shown previously, Tig/Tet treatment caused mitochondrial stress in high metastatic cells, including reduction in ATP levels and higher oxidative stress (Fig 4). The reduction in ATP levels accumulates its precursor AMP, which causes the subsequent phosphorylation of energy sensor AMP kinase for the activation of other protein kinases for survival under bioenergetic stress. Although, ATP levels were mainly decreased in high metastatic cells, we found increased phosphorylation of AMPK (pAMKα-Thr172) in both high and low metastatic cells after treatment (Fig 6B).

Regarding the effect of Tig/Tet treatment on proliferative signaling, reduced levels of AKT phosphorylation and cMyc were observed in high-metastatic cells compared with either unchanged or increased levels in low-metastatic cells (Fig. 6C). Similarly, the phosphorylation of cellular invasion and metastasis mediators, such as FAK and SRC proteins, was also reduced in high-metastatic cells, whereas their expression remained unchanged in low-metastatic cells. In contrast, the expression of apoptotic cell death markers, such as higher caspase-3 and PARP cleavage, was observed in high-metastatic cells after Tig/Tet treatment (Fig 6D). In addition, alternate death pathways, such as autophagy, were also upregulated, as indicated by higher LC3B-II levels and lower p62 levels in high-metastatic cells (Fig 6D).

Altogether, the analysis of signaling pathways clearly indicated that Tig/Tet treatment inhibited mitochondrial biogenesis, proliferative and metastatic signaling, and activated cell death through apoptosis and autophagy in high metastatic cells, contributing to their enhanced sensitivity to Tig/Tet.

### Combined treatment of oxaliplatin with Tig/Tet effectively sensitizes high-metastatic cells

**Fig. 7:**
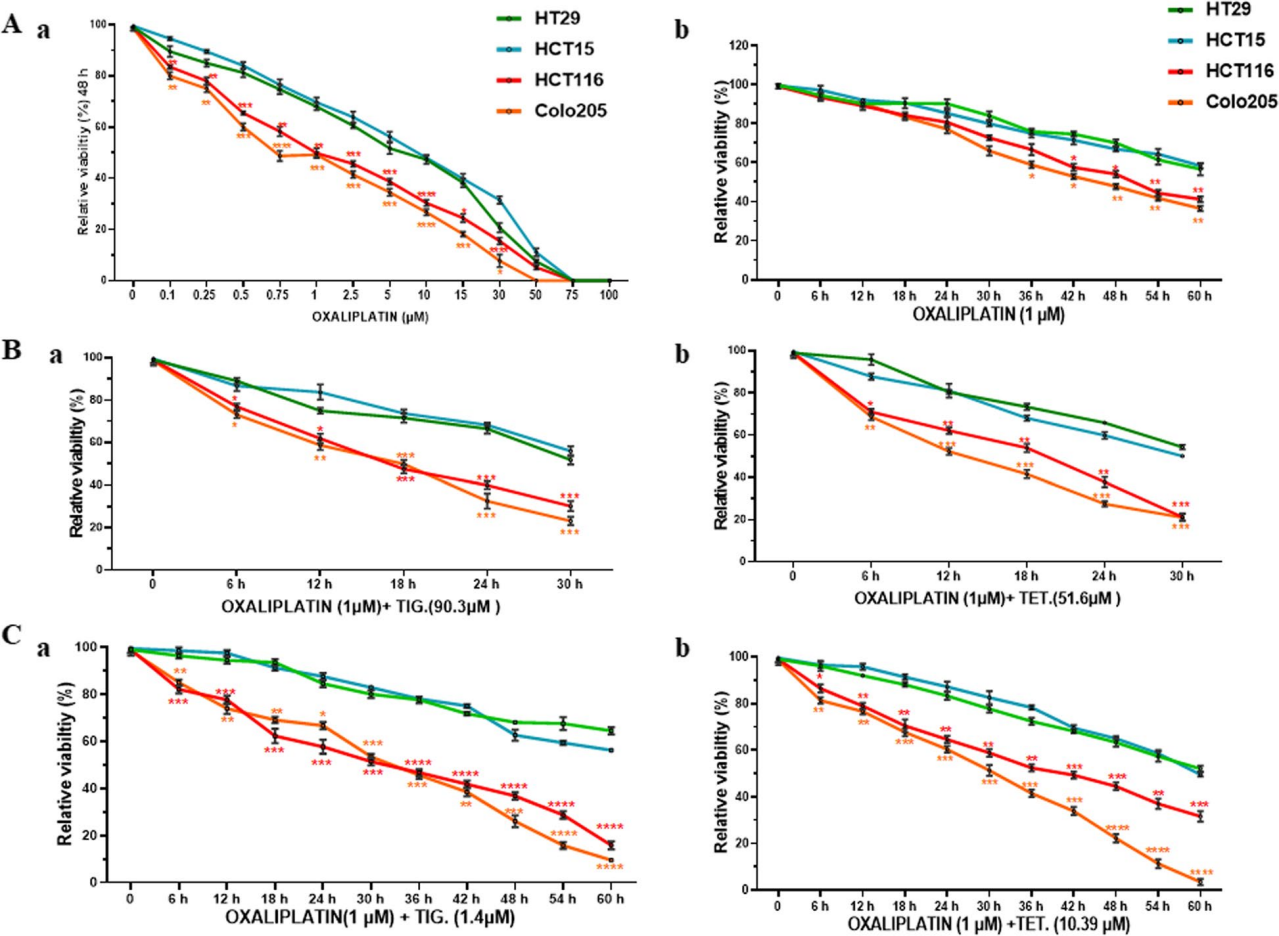
Effect of combined treatment of oxaliplatin with Tig/Tet on the sensitivity of CRC cells. Equal numbers of cells were seeded in 96-well plates (10,000 cells/well) in regular media, and the next day, the media was replaced with media containing different combinations of agents as indicated for varying concentrations/time points. A Graphs represent the relative viability of cells treated with a different concentrations of oxaliplatin for 48h and b treated with 1µM of oxaliplatin for different time points. B Graphs represent the relative viability of the combined treatment of oxaliplatin with a Tig and b Tet at their IC_50_ concentrations for indicated time points. C Similarly, the graphs represent the relative viability of the combined treatment of oxaliplatin with a Tig and b Tet at their physiological concentrations for indicated time points. Percentage viability is presented as relative to non-treated controls, and significant differences in the viability between low and high metastatic cells were calculated using student’s t-test. *P<0.05**P<0.01 and ***P<0.001

To understand whether Tet/Tig could be useful in improving the effectiveness of existing chemotherapeutics, the effect of Tig/Tet with the well-known third-generation platinum drug oxaliplatin on the sensitivity of CRC cells was measured. Initially, to test the efficacy of oxaliplatin and to determine the IC_50_ concentration, CRC cells were treated with an increasing dosage of oxaliplatin for 48h and viability was measured using a trypan blue assay. As shown in Fig 7Aa, high-metastatic cells were more sensitive to oxaliplatin treatment (IC_50_ <1µM), compared to the low-metastatic cells (IC_50_=4.8-6.8 µM) (Supplementary Table 2). Similarly, treatment with oxaliplatin at a dose close to the IC_50_ of high-metastatic cells (1µM), showed a significant difference in their viability compared with low-metastatic cells with increasing time points, including approximately 50% and 40% reduction at 48 and 60 h, respectively (Fig 7Ab). To measure the combinatorial effect, the cells were treated with oxaliplatin (1µM) with Tig/Tet at their IC_50_ concentrations, and viability was measured at different time points. Although the viability decreased after the combined treatment in all CRC cells, the high metastatic cells were more sensitive to treatment as early as 6 h until 30 h (Fig. 7Ba-b). Interestingly, this combined treatment of oxaliplatin with Tig/Tet at their respective IC_50s_ was more effective and caused an approximately 50% reduction in the viability of high metastatic cells as early as 18h compared to 48h of standalone treatment with oxaliplatin (Fig. 7Ab and Ba-b). To test the efficacy of this combined treatment at physiological concentrations, cells were treated with oxaliplatin and serum equivalent concentrations of Tig (1.4 µM)/Tet (10.39 µM), as reported earlier [20, 21], for different time points and viability measurements. Similar to their IC_50_ concentrations, we found that Tig/Tet treatment at their physiological concentrations with oxaliplatin was also effective in sensitizing high-metastatic cells as early as 6h (Fig. 7Ca-b). However, in this case, a 50% reduction in the viability of high metastatic cells was observed at later time points, such as 30 h (for Tig) and 30–36 h (for Tig) (Fig 7Ca-b). Altogether, the combined treatment of oxaliplatin with Tig/Tet was more effective in sensitizing high metastatic cells at early time points compared with low metastatic cells.

## Discussion

Approximately 20-50% of locally advanced CRC cases develop metastasis to other organs such as the liver and lungs, which contributes to most CRC-related deaths [21]. In terms of the treatment of metastatic CRC, a combination of systemic therapy and curative surgery has been suggested to improve the survival rate [22]. Although early therapeutic intervention is the key to improving response rate and patient survival, drug resistance and adaptation to carcinogenic insult by cancer cells remain a major challenge in regulating CRC metastatic progression [23].

Enhanced glycolysis aids cancer cells through rapid ATP generation (Warburg effect); however, most cancer cells retain or even enhance OXPHOS for the supply of macromolecules and survival under hypoxic conditions [24, 25]. Mitochondrial alterations, including mtDNA copy number, biogenesis, OXPHOS, and free radical signaling, have been reported in different cancers and are also known to contribute to the hallmark of cancer metastasis [26]. However, the mechanistic role and therapeutic targeting of mitochondria in metastatic CRC remain unclear.

Previously, different classes of antibiotics, including anthracyclines and non-anthracyclines, have been investigated for their ability to inhibit cancer cell proliferation [11]. Among them, bacterial translational inhibitor antibiotics could inhibit proliferation by targeting overdriven mitochondria because of the conserved similarities between mitochondrial and bacterial ribosomes. Our results on the viability of low- and high-metastatic CRC cells after treatment with these different antibiotics showed dose-dependent inhibition across these CRC cells. However, among them, high-metastatic cells were more sensitive to the small subunit inhibitors Tig/Tet and showed significant inhibition in viability with lower IC_50_ values (with less than half of the IC_50_ values compared to low-metastatic cells). Therefore, we selected these antibiotics and further investigated their potential modes of action in sensitizing high-metastatic cells.

Tetracycline and its derivatives, such as doxycycline, exhibit cytotoxic effects in various cancer cells [12, 27, 28]. Similarly, the tumor inhibitory role of tigecycline has recently been investigated in different cancers [29]. Our study found not only a significant inhibition in the viability of high metastatic cells upon Tig/Tet treatment but also their ability to limit cell migration/invasion as well as non-adherent spheroid formation, which are characteristic features of metastatic progression. Because Tig and Tet bind to mitochondrial 16s rRNA subunits, interfering in mitochondrial biogenesis and/or functions could be the possible growth inhibitory mechanism of Tig/Tet in cancer cells [9, 12]. Therefore, analysis of mitochondrial biogenesis, functions, RC assembly, and activity after Tig/Tet treatment could allow further understanding of their anti-proliferative activities. As indicated by reduced mtDNA copy number and biogenesis markers (PGC1/NRF-1/NRF-2 and TFAM) after Tig/Tet treatment, our results indicated that these antibiotics interfere with mitochondrial content and biogenesis specifically in high-metastatic cells. In the mitochondrial biogenesis pathway, PGC1-α co-activates NRF1/NRF2 and further activates TFAM, which is required for mtDNA replication and controls the mtDNA copy number and expression of RC subunits [30]. Since, synergetic expression of these biogenesis markers increases mitochondrial numbers and respiratory activity, therefore, in our study, the downregulation of PGC1α and TFAM in high-metastatic cells after Tg/Tet treatment could be linked to a significant decline in mitochondrial functions, as observed in these cell lines after treatment. Since, PGC1α and TFAM are nuclear encoded, this further indicates that these antibiotics may interfere with nuclear transcription and translation in high-metastatic cells.

In contrast, an increase in the mtDNA copy number/biogenesis markers in low metastatic cells was observed after Tig/Tet treatment. The exact reason for this increase in mtDNA copy number/biogenesis is unclear, published reports suggest that the threshold of mtDNA copy number is required to maintain cellular homeostasis [31]. As discussed earlier, generally, an increase in the mtDNA copy number is accompanied by coordinated upregulation of upstream mitochondrial biogenesis factors (PGC1-alpha, NRF-1, and -2) and mtDNA replication machinery (TFAM) [30, 32]. Therefore, changes in mtDNA copy number and biogenesis markers indicate mitochondrial numbers or the requirement of mitochondria for cellular homeostasis. Specifically, an increase in mtDNA/biogenesis is a compensatory mechanism of survival under stress or limiting factors, including ATP and nucleotide, and also to overcome the effect of pathogenic mtDNA mutations such as oxidative stress [31, 33, 34]. Conversely, low mtDNA copy number increases the sensitivity of tumor cells to chemotherapeutic drugs, which is associated with increased ROS levels [35].

In our study, the survival of low metastatic cells was significantly higher than that of high metastatic cells after antibiotic treatment (Fig. 1). In addition, subsequent assessment suggested that upon antibiotic treatment, mitochondrial functions (ROS, superoxides, membrane potential and ATP levels) remain mostly unaltered in low metastatic cells (Fig 4). Therefore, it is most likely that in response to Tig/Tet stress, low metastatic cells maintained the viability and mitochondrial functionality for their survival by supplying fresh mitochondria, which was reflected by a corresponding increase in mitochondrial number/biogenesis markers in these cells (especially at 48h of Tg/Tet treatment) (Fig. 3). In contrast, in high-metastatic cells, which are known to rely heavily on mitochondrial functions for proliferation and metastatic potential [13], mitochondria of these cells were inhibited by Tig/Tet treatment. Therefore, the sensitivity of high-metastatic cells to antibiotic treatment could be due to the failure to increase the mtDNA number/biogenesis pathway and/or mitochondrial inhibition, including enhanced oxidative stress and ATP depletion in these cells.

The Tig/Tet class of antibiotics has been independently reported for their inhibitory role in mitochondrial functions and protein synthesis [36, 37]. However, limited information is available on their effects on CRC cells. For example, Dijk et al. showed that doxycycline (a tetracycline) treatment increases ROS, decreases mitochondrial proteins and functions, and sensitizes lung cancer cells to apoptosis with gemcitabine [36]. Similarly, chemically modified tetracyclines (CMTs) induce both apoptosis and necrosis in metastatic prostate cancer cells, with increased ROS levels, mitochondrial depolarization, and cell migration inhibition [37]. Tigecyclin also inhibits the translation of mitochondrial proteins, the activities of RC complexes, and mitochondrial respiration in renal carcinoma cells [38]. In addition, tigecycline preferentially sensitizes cells with increased mitochondrial translation, such as *K-Ras* mutant CRC cells and leukemia stem and progenitor cells, compared with their normal counterparts [39, 40]. Our findings are in agreement with these reports and further indicate that Tig/Tet treatment could be more effective in sensitizing high-metastatic CRC cells and targeting their mitochondrial activity. Specifically, we found that Tig/Tet treatment caused a significant decline in mitochondrial functions by enhancing oxidative stress and membrane depolarization and reducing ATP content in high-metastatic cells. Such a decline in function could be due to the downregulation of overall mitochondrial protein synthesis, as observed by BN-PAGE analysis after treatment in these cells. More specifically, we found that C-I assembly and activity were severely compromised after Tig/Tet treatment in high metastatic cells. Because C-I is the largest among RC complexes encoded by both nuclear and mitochondrial genes [41], the interaction between Tig/Tet and C-I subunits and their specific mechanism of inhibition remain to be investigated.

Apart from mitochondrial inhibition, the anticancer activity of Tig/Tet has been associated with their ability to inhibit the proliferative pathway and/or induce cell death mainly through apoptosis. For example, CMT-3 or doxycycline treatment inhibits the release of matrix matello-proteases and enhances apoptotic bodies in prostate cancer cells [37]. Similarly, tigecycline limits cell growth by inhibiting proliferative/survival pathways such as Myc/HIFs, PI3K/AKT, AMPK signaling, p21CIP1/Waf1 and Wnt/β-catenin signaling pathways [29]. After mitochondrial inhibition, we also found that Tig/Tet treatment significantly inhibited the proliferative pathways (lower pAKT and cMYC) and downregulated the metastatic signaling (reduced SRC and FAK phosphorylation) in high-metastatic cells. In contrast, significant cleavage of caspase-3 and PARP and activation of autophagy, were observed, indicating the dual mode of cell death by these antibiotics in high-metastatic CRC cells. Altogether, our study in CRC cells indicates that mitochondrial inhibition caused by Tig/Tet treatment downregulates survival pathways and induces cell death in high-metastatic cells.

Because these antibiotics are already in clinical use for treating bacterial infections, their nonbacterial activities with chemotherapy/targeted therapy have been exploited to enhance the susceptibility of cancer cells. However, their standalone or combinatorial effects vary among different cancer types. For example, doxycycline treatment partially inhibits mitochondrial translation and alone has no effect on apoptosis; however, in combination with gemcitabine, it increases caspase activity and reduces the viability of lung cancer cells [36]. In contrast, tigecycline significantly inhibits the growth, colony formation, and survival of RCC cells and further enhances its efficacy when combined with paclitaxel [42]. The synergistic action of these antibiotics increases the sensitivity of tumor cells (pancreatic and ovarian cancers) and regulates tumor growth in both in vitro and in vivo models [43, 44]. In line with these previous reports, we also found that the synergistic effect of Tig/Tet with the standard drug oxaliplatin was more effective in preferentially sensitizing high-metastatic cells not only at their IC_50_ concentrations but also at their physiological concentrations. Altogether, Tig/Tet treatment selectively sensitizes high-metastatic cells by targeting mitochondrial pathways and may be used synergistically with chemotherapeutic agents at clinically relevant concentrations to treat tumors with high mitochondrial activities.

Although the study was straightforward, its limitations are worth mentioning. Because both of these antibiotics are FDA-approved agents and have been well tolerated in normal cells even at higher concentrations, we investigated their effect only in CRC cells and did not utilize normal colon cells, which could have provided further information on their effect on non-cancer cells. Because both sets of metastatic cells (low and high) were previously characterized for their differences in their metastatic potential and mitochondrial functions [13], the major focus of the present study was to target the increased mitochondrial activities of high-metastatic cells relevant to metastatic potential. Therefore, we preferentially investigated the mechanism of increased sensitivity of high-metastatic cells toward Tig/Tet. Our study also lacks in vivo validation of Tig/Tet sensitivity in CRC metastasis. Although Tig/Tet has been successfully shown to reduce tumor growth in vivo in various xenograft models, including those derived from prostate, renal, and gastric cancer cells [37, 42, 45], the lack of in vivo investigation, especially using an immune-competent CRC animal model, remains a major limitation of these studies, including ours. However, a recent report on CRC has shown that tigecycline treatment inhibits tumorigenesis both in vitro and in a colitis-associated colorectal cancer (CAC) murine model [46]. This tumor inhibitory effect is mediated via inhibition of Wnt/β-catenin pathway, apoptosis induction, and reduction of the cancer immune response in the CAC model. Our findings further suggest that metastatic conditions could be regulated by targeting mitochondrial functions and inducing apoptotic cell death pathways. However, further studies are required to independently investigate the effect on Tig/Tet in such CAC models of CRC to find any overlap between classical Wnt/β-catenin pathway and mitochondrial driven pathways.

## Conclusion

**Fig. 8:**
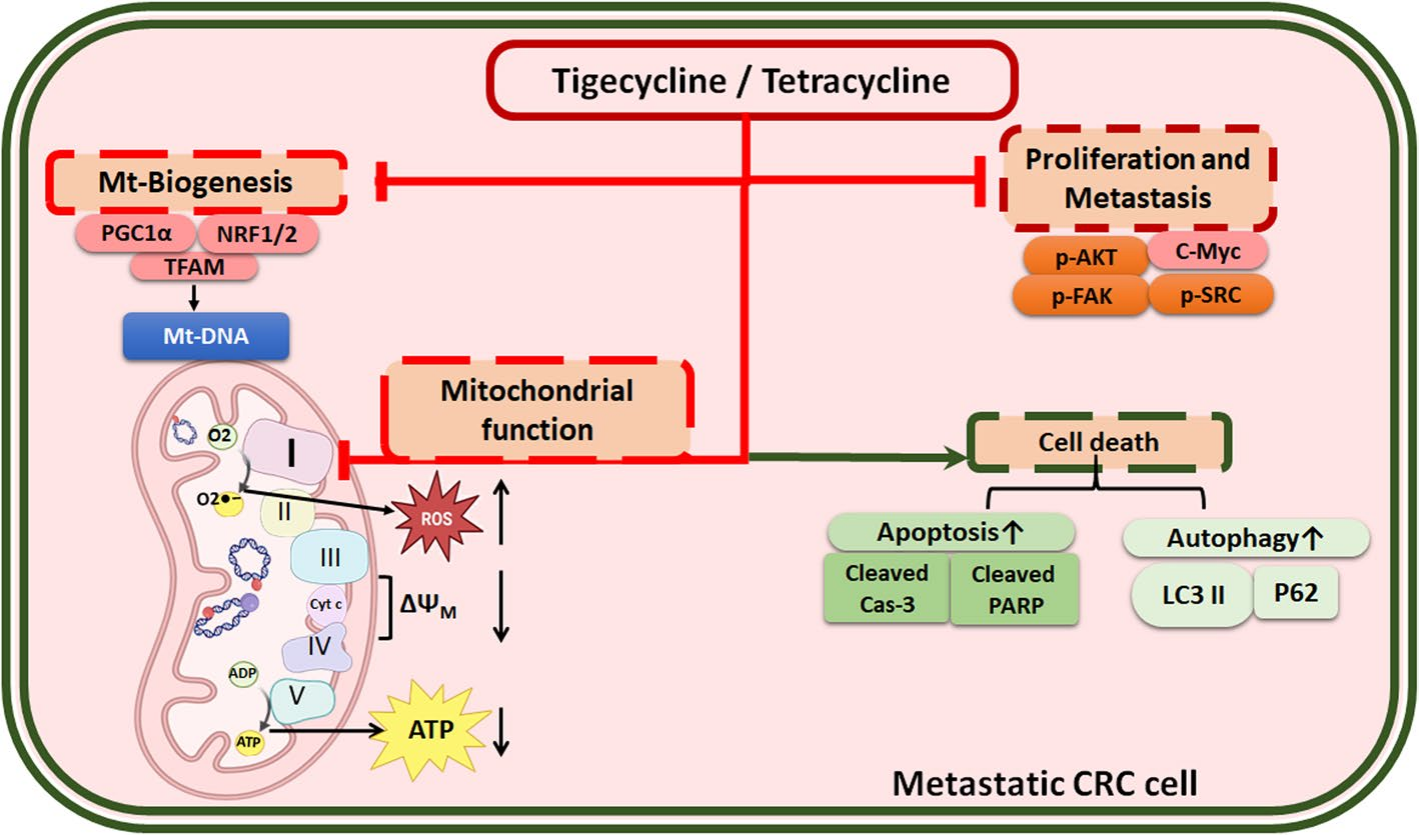
The figure summarizes the potential mechanism of action of mitochondrial antibiotics in high metastatic cells via inhibition of mitochondrial pathways (biogenesis and functions) and proliferative & metastatic signaling, as well as activation of the cell death pathways. Figure is prepared using icons “Mitochondria, Electron Transport Chain”, by BioRender.com (2023). Abbreviations: Mt, Mitochondrial; NRF1. nuclear respiratory factor 1; PGC1-α, peroxisome proliferator-activated receptor-gamma coactivator (PGC)-1alpha; TFAM, transcription factor A, mitochondrial; p-, phosphorylated; AKT, protein kinase B; FAK, focal adhesion kinase, SRC, tyrosine-protein kinase Src; PARP, Poly (ADP-ribose) polymerases; LC3B, microtubule associated protein 1 light chain 3 beta; ROS: reactive oxygen species, ΔΨM: mitochondrial membrane potential, ATP: adenosine triphosphate; CRC, colorectal cancer

Altogether, our study clearly demonstrated that Tig/Tet treatment a) inhibits mitochondrial biogenesis, functions, more specifically C-I assembly and activity, and b) downregulates proliferative signaling and upregulates apoptotic cell death, which together sensitize high-metastatic cells (Fig. 8). Our results are consistent with general inferences regarding mitochondrial inhibition by tigecycline/tetracycline. However, our study is unique in addressing their inhibitory effects specifically in regulating the high-metastatic potential of CRC cells by showing a general mechanism of inhibition of mitochondrial biogenesis and functions and a specific mechanism of inhibition of C-I assembly and activity in high-metastatic CRC cells. Given their existing clinical use, Tig/Tet could be repurposed to regulate cancer growth, particularly in mitochondrial-driven metastatic cancers. It also provides therapeutic opportunities to explore potential combinations with existing cancer drugs to improve the efficacy of treatment.

### Supplementary Information

**Supplementary Material 1.**

**Supplementary Material 2.**

## Data Availability

The data analyzed in this study are available from the corresponding author upon reasonable request.
