# Supplementary material for: Regulation of metastatic potential by drug repurposing and mitochondrial targeting in colorectal cancer cells
Source: BMC Cancer. 2024 Mar 8;24:323. doi: 10.1186/s12885-024-12064-5 (PMC10921801; doi:10.1186/s12885-024-12064-5)

• **Uncropped Gels/Western blots of Fig.5**

A .a. BN-PAGE Gel

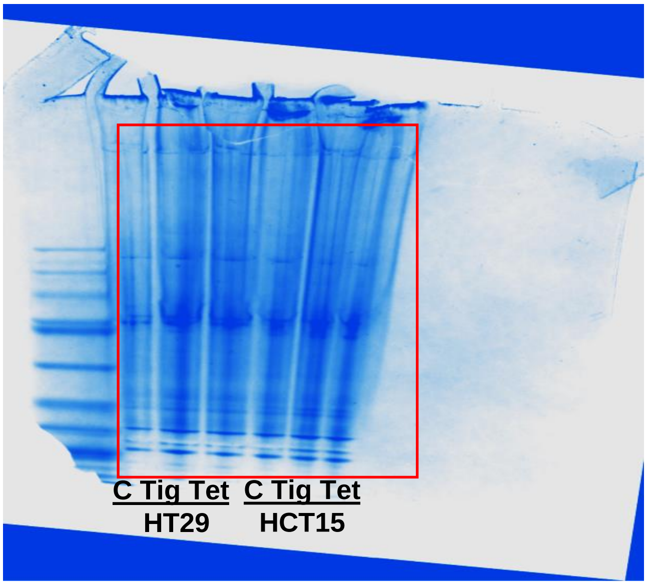

B. a. BN-PAGE Gel

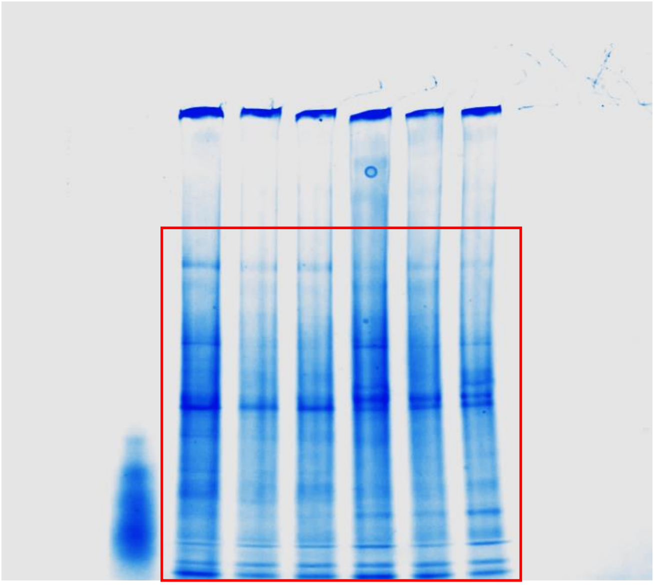

Western blot of loading control (VDAC)

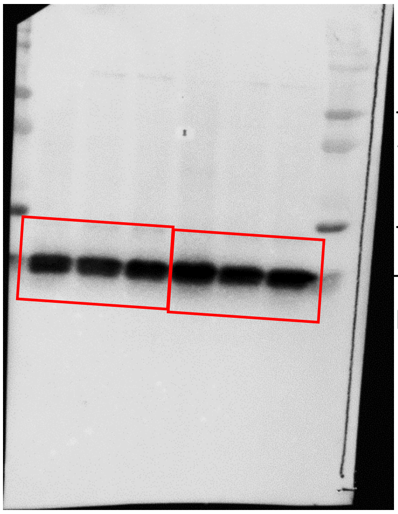

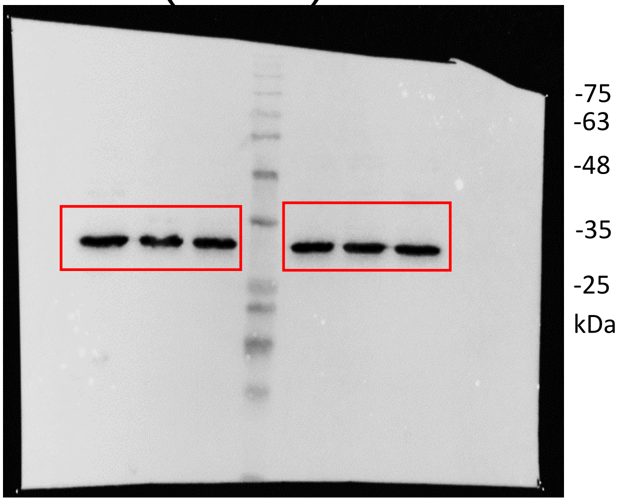

B (e) Uncropped Gels of In-Gel Assays

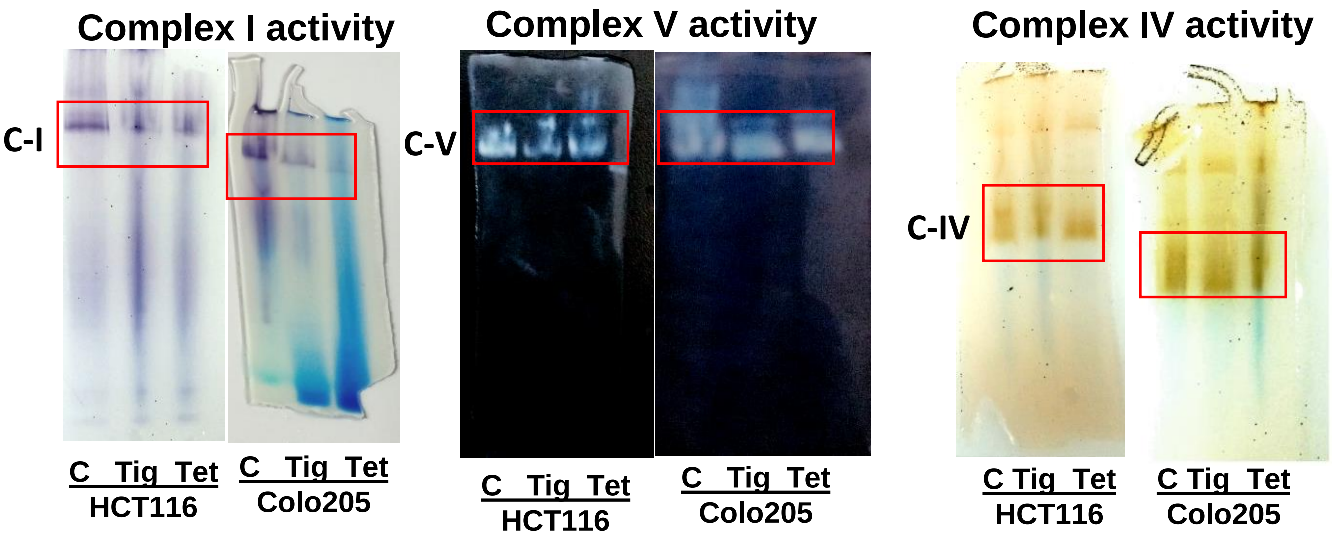

Western blot of loading control (VDAC)

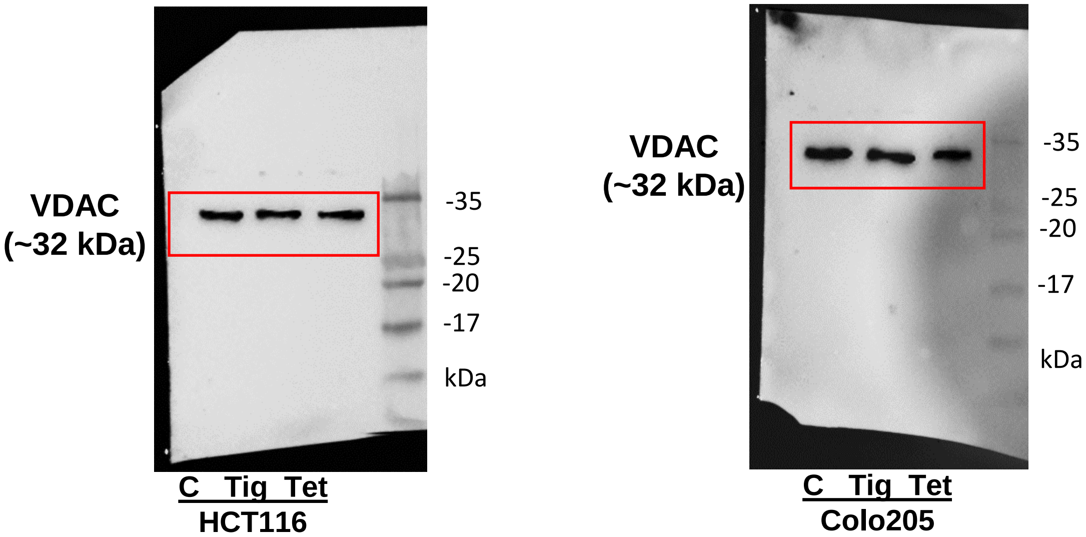

• **Uncropped Western blots of Fig. 6**

A .Mitochondrial Biogenesis

PGC1 α
(~ 32 kDa)

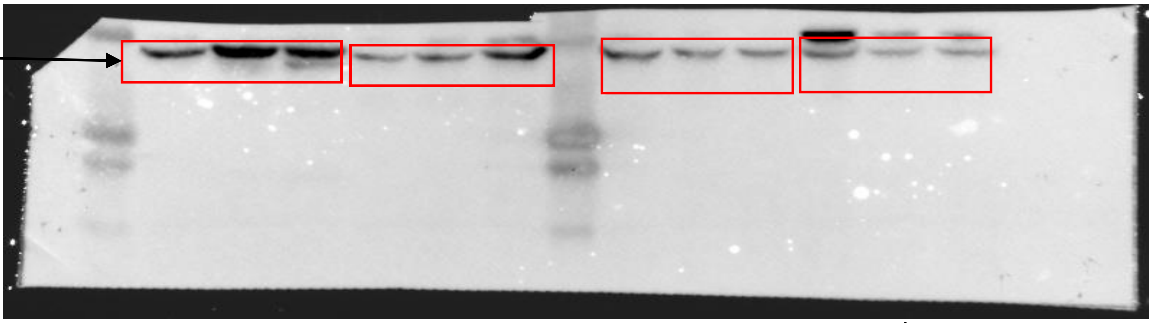

-35
-25
-19
kDa

C Tig Tet
HT29
C Tig Tet
HCT15
C Tig Tet
HCT116
C Tig Tet
Colo205

TFAM
(~29 kDa)

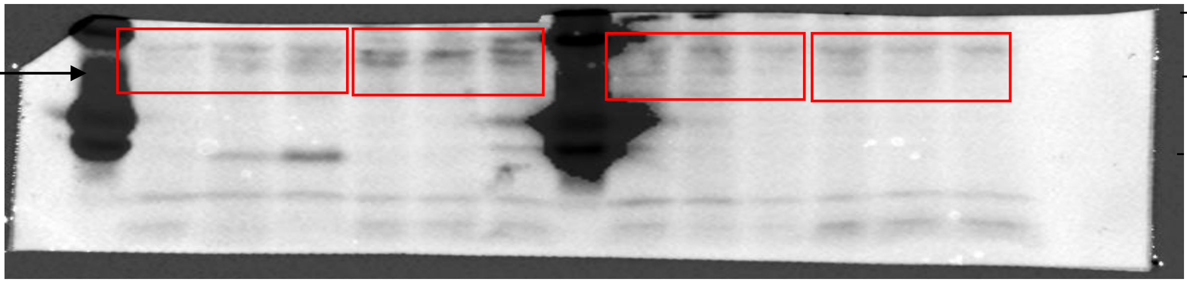

-35
-25
-19
kDa

Exposure-18 sec

C Tig Tet
HT29
C Tig Tet
HCT15
C Tig Tet
HCT116
C Tig Tet
Colo205

TFAM
(~29 kDa)

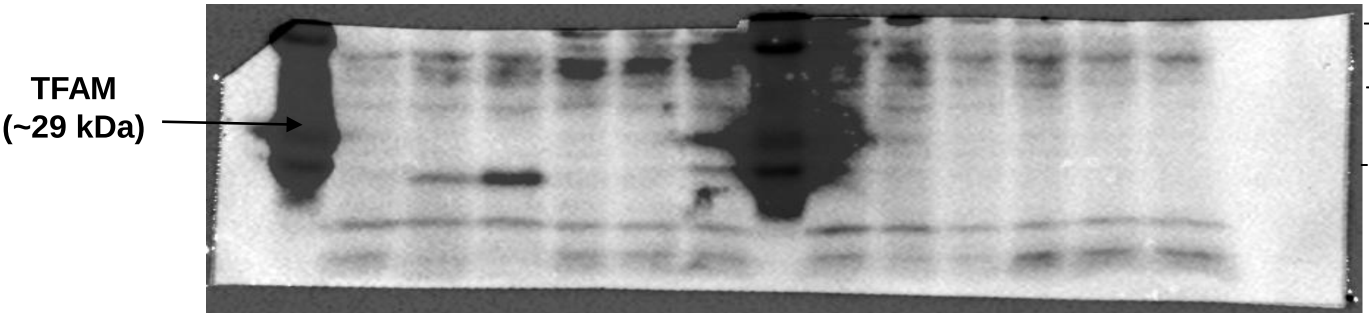

-35
-25
-19
kDa

Exposure-36.6 sec

C Tig Tet
HT29
C Tig Tet
HCT15
C Tig Tet
HCT116
C Tig Tet
Colo205

β-Actin
(~43kDa)

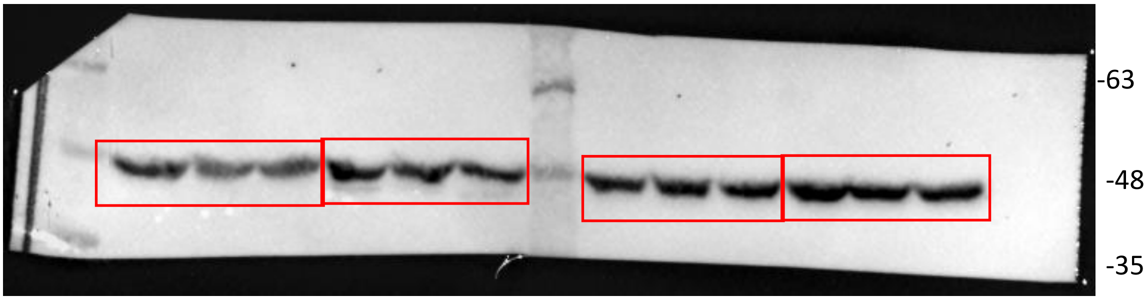

-63
-48
-35

C Tig Tet
HT29
C Tig Tet
HCT15
C Tig Tet
HCT116
C Tig Tet
Colo205

B. Energy Sensing

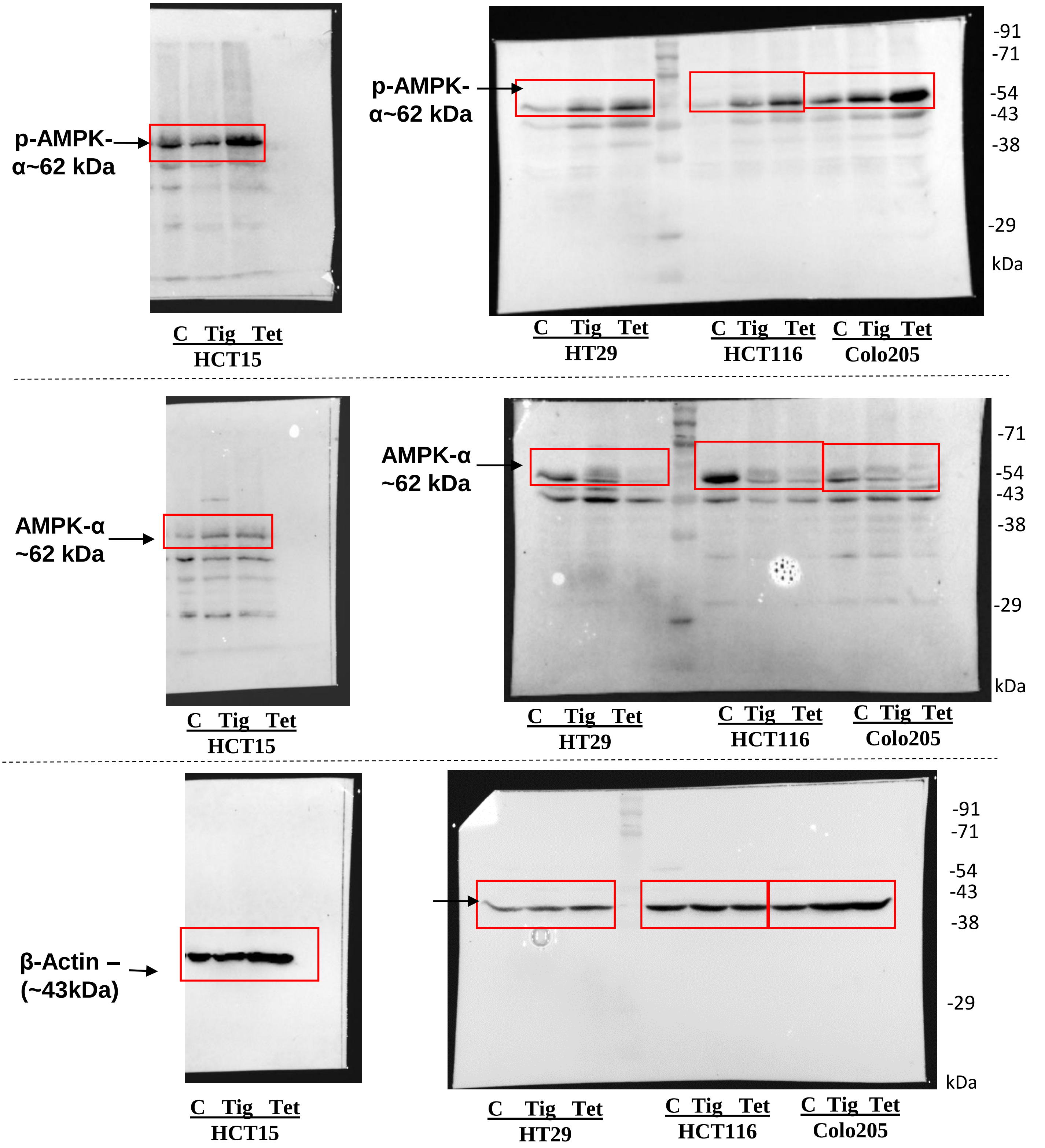

C. Cell Proliferation and metastatic potential

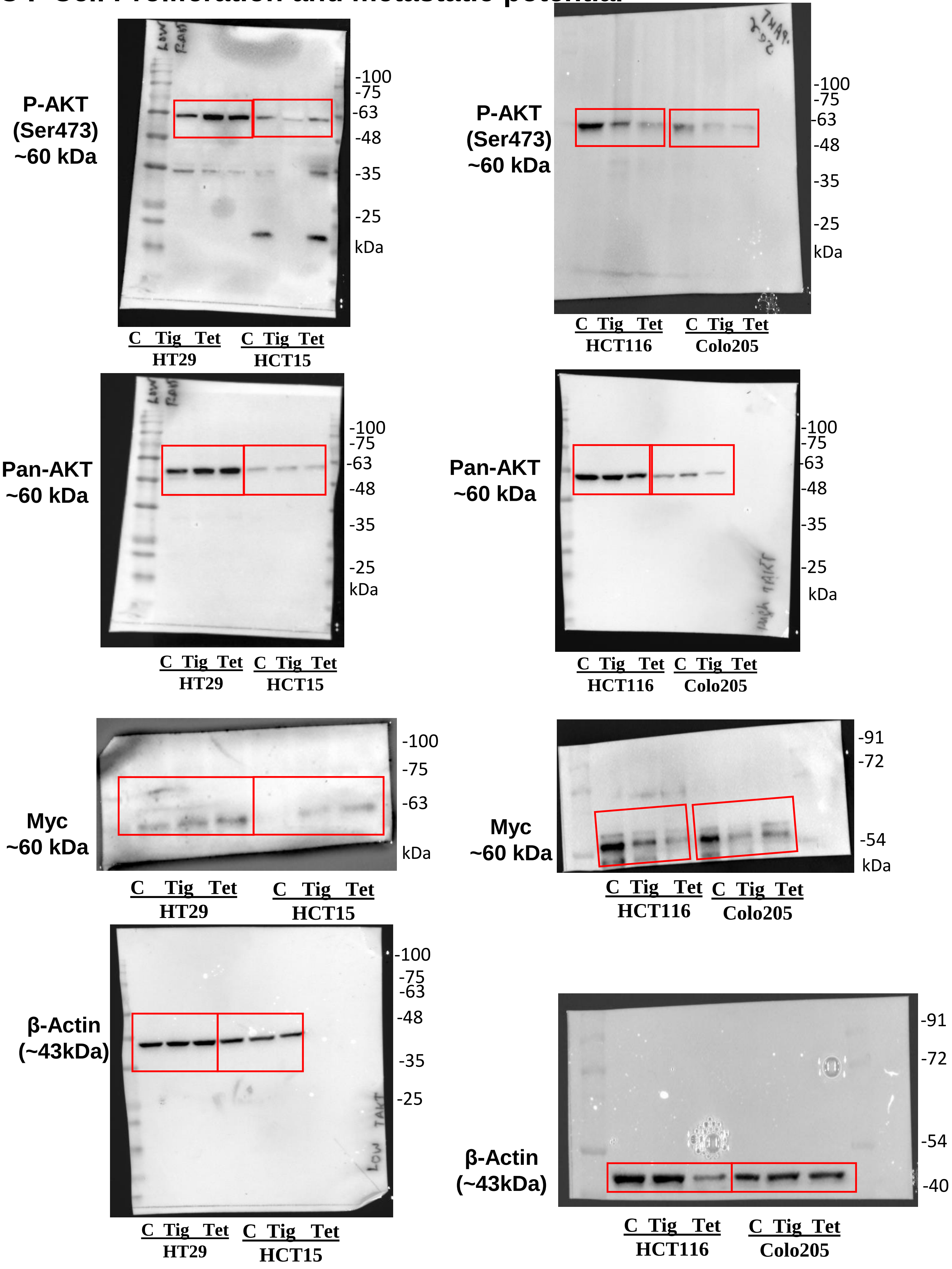

Continued….

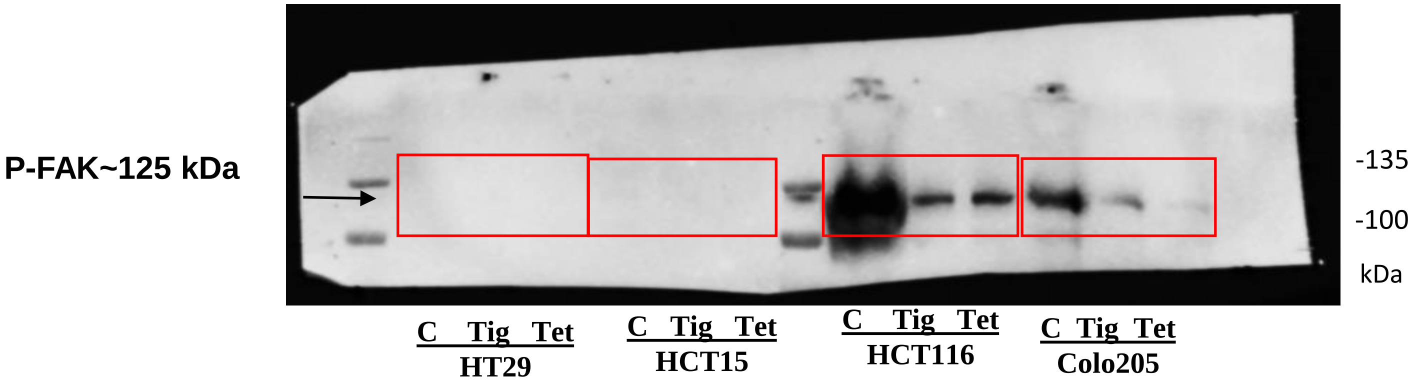
P-FAK~125 kDa
-135
-100
kDa
C Tig Tet
HT29
C Tig Tet
HCT15
C Tig Tet
HCT116
C Tig Tet
Colo205

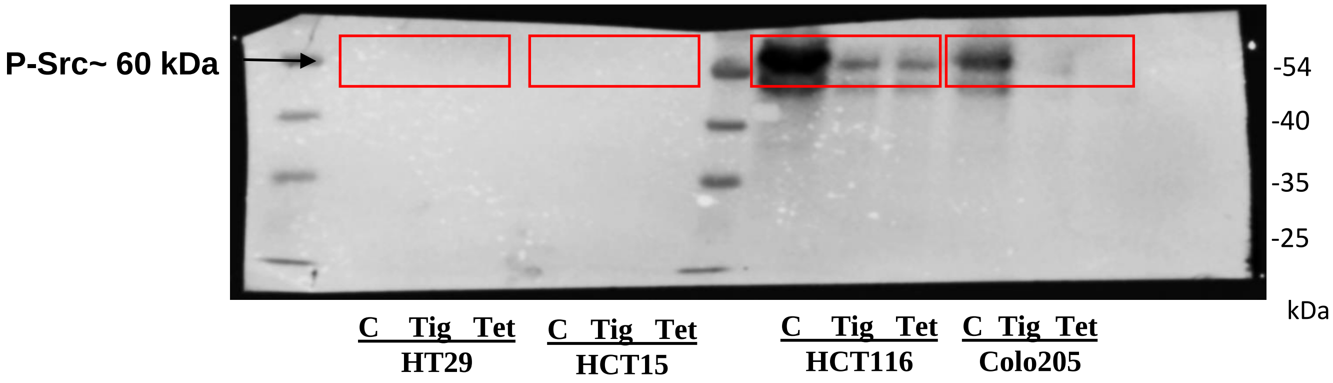
P-Src~ 60 kDa
-54
-40
-35
-25
kDa
C Tig Tet
HT29
C Tig Tet
HCT15
C Tig Tet
HCT116
C Tig Tet
Colo205

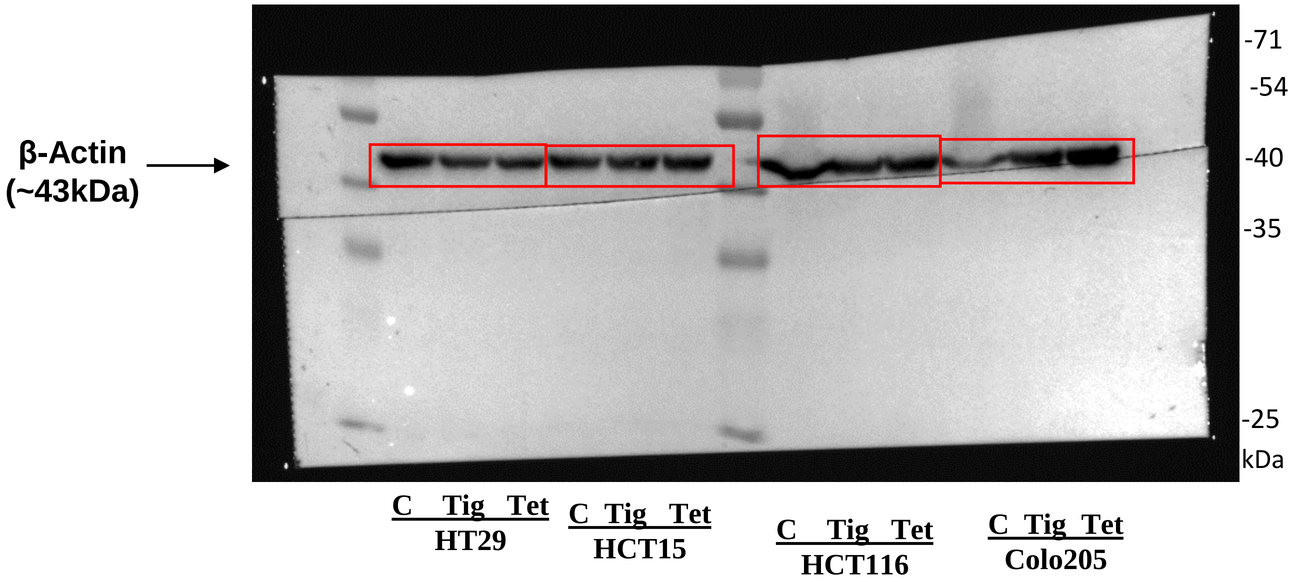
β-Actin
(~43kDa)
-71
-54
-40
-35
-25
kDa
C Tig Tet
HT29
C Tig Tet
HCT15
C Tig Tet
HCT116
C Tig Tet
Colo205

D． Death Pathways (Apoptosis)

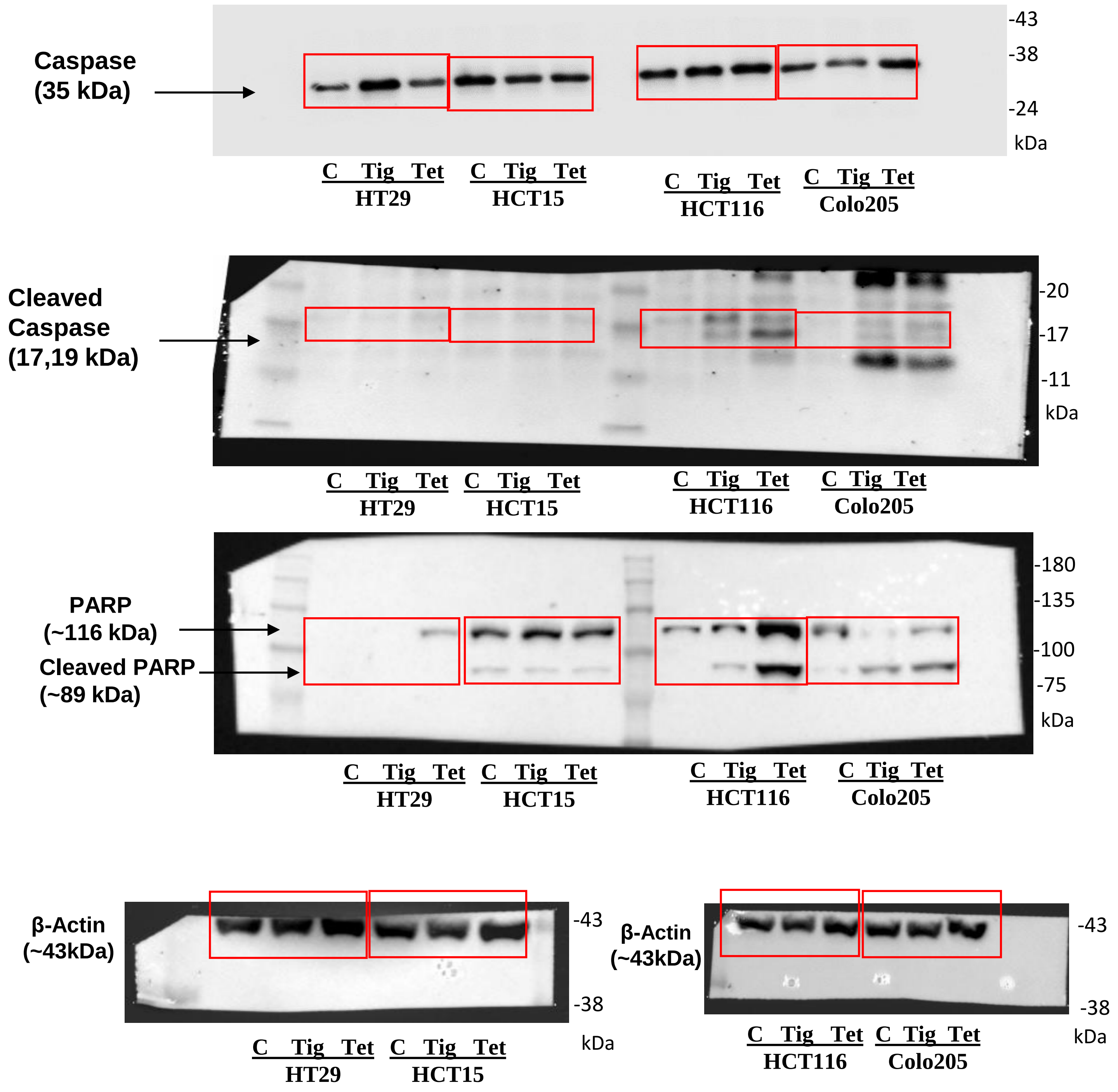

Continued …..Death Pathways (Autophagy)

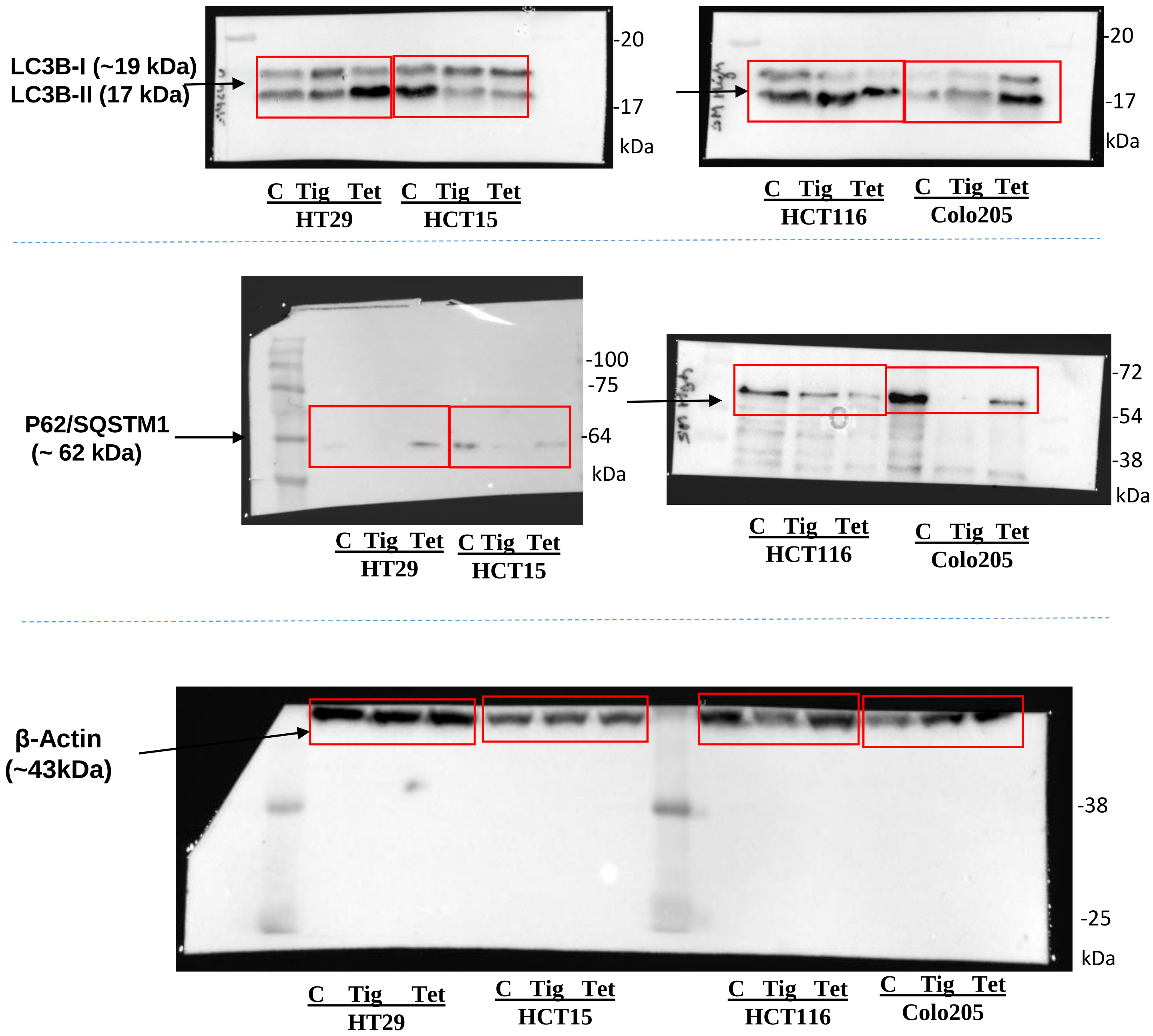

Supplement: Supplementary file 1 — Supplementary Material 1. [file 12885_2024_12064_MOESM1_ESM.pdf]
