# Supplementary material for: Regulation of metastatic potential by drug repurposing and mitochondrial targeting in colorectal cancer cells
Source: BMC Cancer. 2024 Mar 8;24:323. doi: 10.1186/s12885-024-12064-5 (PMC10921801; doi:10.1186/s12885-024-12064-5)
Supplement: Supplementary file 2 — Supplementary Material 2. [file 12885_2024_12064_MOESM2_ESM.docx]

**Supplementary information:**

**Supplementary Table -1:** IC_50_ values of tigecycline and tetracycline treatment in CRC cells at 48 h

| **Metastatic potential** | **Cells** | **Tigecycline (Tig)**  **IC_50_ (µM±SD)** | | **Tetracyclin (Tet)**  **IC_50_ (µM±SD)** | |
| --- | --- | --- | --- | --- | --- |
| Low | HT29 | 201.2 ± 19.3 | Mean  196.35 ±17.4 | 108.6 ± 12.4 | Mean  102.5 ± 15.8 |
|  | HCT15 | 191.5 ± 15.4 |  | 96.5 ± 18.4 |  |
| High | HCT116 | 97.2 ± 26.3 | Mean  90.3 ± 23.06 | 53.2 ± 20.5 | Mean  51.5 ± 19 |
|  | Colo205 | 83.4± 19.8 |  | 49.8 ± 17.5 |  |

**Supplementary Table -2:** IC_50_ values of oxaliplatin treatment in CRC cells at 48 h

| **Metastatic potential** | **Cells** | **Oxaliplatin**  **IC_50_ (µM)** |
| --- | --- | --- |
| Low | HT29 | 4.82 ± 0.029 |
|  | HCT15 | 6.85 ±0.011 |
| High | HCT116 | 0.86 ±0.23 |
|  | Colo205 | 0.74 ± 0.31 |
